# Is Cadmium Toxicity Tissue-Specific? Toxicogenomics Studies Reveal Common and Specific Pathways in Pulmonary, Hepatic, and Neuronal Cell Models

**DOI:** 10.3390/ijms23031768

**Published:** 2022-02-04

**Authors:** Matilde Forcella, Pierre Lau, Marco Fabbri, Paola Fusi, Monica Oldani, Pasquale Melchioretto, Laura Gribaldo, Chiara Urani

**Affiliations:** 1 Department of Biotechnology and Biosciences, University of Milano-Bicocca, Piazza della Scienza 2, 20126 Milano, Italy; matilde.forcella@unimib.it (M.F.); paola.fusi@unimib.it (P.F.); m.oldani12@campus.unimib.it (M.O.); 2 Joint Research Centre (JRC), European Commission, 21027 Ispra, Italy; laupierre008@gmail.com (P.L.); marco.fabbri@ec.europa.eu (M.F.); 3 Department of Earth and Environmental Sciences, University of Milano-Bicocca, Piazza della Scienza 1, 20126 Milan, Italy; pasquale.melchioretto@unimib.it

**Keywords:** cadmium, cancer, metal homeostasis, neurotoxicity, toxicogenomics

## Abstract

Several harmful modifications in different tissues-organs, leading to relevant diseases (e.g., liver and lung diseases, neurodegeneration) are reported after exposure to cadmium (Cd), a wide environmental contaminant. This arises the question whether any common molecular signatures and/or Cd-induced modifications might represent the building block in initiating or contributing to address the cells towards different pathological conditions. To unravel possible mechanisms of Cd tissue-specificity, we have analyzed transcriptomics data from cell models representative of three major Cd targets: pulmonary (A549), hepatic (HepG2), and neuronal (SH-SY-5Y) cells. Further, we compared common features to identify any non-specific molecular signatures. The functional analysis of dysregulated genes (gene ontology and KEGG) shows GO terms related to metabolic processes significantly enriched only in HepG2 cells. GO terms in common in the three cell models are related to metal ions stress response and detoxification processes. Results from KEGG analysis show that only one specific pathway is dysregulated in a significant way in all cell models: the mineral absorption pathway. Our data clearly indicate how the molecular mimicry of Cd and its ability to cause a general metal ions dyshomeostasis represent the initial common feature leading to different molecular signatures and alterations, possibly responsible for different pathological conditions.

## 1. Introduction

Cadmium (Cd) is an environmental pollutant of global concern. It is the seventh substance in the priority list of the Agency for Toxic Substances and Disease Registry (USA) and it is comprised within the first batch of elements identified in the “12th Five Year Plan on Heavy Metal’s Comprehensive Prevention and Control” in China [1,2]. Cd is a natural element in the Earth’s crust and is released in the environment by natural and anthropogenic activities since it has found an extensive use in industry and agriculture. It is released in the air, land and water leading to environmental pollution and exposure of organisms and humans.

The World Health Organization’s guideline set to 3 µg/L the threshold value in drinking water, and the Joint FAO/WHO Expert Committee on Food Additives established a provisional tolerable monthly intake of 25 μg/kg body weight, further restricted by the EFSA Panel on Contaminants in the Food Chain which nominated a tolerable weekly intake of 2.5 μg/kg body weight to ensure sufficient protection of all consumers [3].

For non-occupationally exposed individuals and for non-smokers, the diet through contaminated food and water, is a major source of Cd [4]. The mean dietary intake of Cd is estimated to be 2.3 µg/kg body weight per week. After absorption, Cd is bound to metallothioneins in the liver, organ where it shows the highest accumulation with the kidneys [5]. Another important way of Cd exposure is represented by inhalation of dust and particulate matter [2], thus leading the lung to be another critical target of Cd exposure with reported concentrations of 0.9–6 µM Cd accumulated in this organ. In the aortic wall of heavy smokers Cd can deposit at concentrations up to 20 µM [6]. In addition, due to inhalation, Cd can be uptaken from the nasal mucosa, transported along the primary olfactory neurons to their terminations in the olfactory bulbs bypassing the intact blood brain barrier, and reaching the brain [7].

Once absorbed, Cd is retained in the human body with a long biological half-life of 10–30 years [5], due to very low excretion rates.

A large amount of literature has been published since the outbreak of Itai-Itai disease in 1912 in Japan, due to Cd release into Jinzu River and consumption of rice grown in Cd-contaminated irrigation water. People affected by the disease showed a wide range of symptoms, mainly related to the skeletal system, and complications such as anemia and kidney failure leading to death [8].

Since then, a wide range of Cd effects has been reported targeting different tissues and organs. Cd is a recognized Group I human carcinogen by the International Agency for Research on Cancer, and occupational and environmental exposure are related to cancers in different organs (e.g., lung, breast, liver, urinary bladder, prostate, pancreas, and nasopharynx). Exposure to Cd may lead to kidneys, liver, skeletal and cardiovascular system damage, and to sight and hearing deterioration, along with effects on the reproductive system during development and adulthood [9]. In addition, more recently, Cd has been associated to neurodegenerative disorders such as amyotrophic lateral sclerosis and Parkinson’s disease [7,10,11].

At the cell and molecular level multiple mechanisms have been recognized in different cell targets of Cd toxicity, such as oxidative stress, interference with essential metals, epigenetic effects [10]. However, despite the increasing knowledge and continuous interest on Cd, the complex mechanisms have not been fully understood and tissue-specificity or more broad and common effects in different targets not fully identified.

To further deepen the understanding of Cd mechanisms and specificity, we have performed a comparison of transcriptomics data from cell models representative of three major Cd targets: pulmonary (A549), hepatic (HepG2), and neuronal (SH-SY-5Y) cells.

In this work we aimed at identifying specific pathways and common features in the three cell models to understand possible tissue-organ specificities and the molecular signatures of this metal.

## 2. Results

To identify whether Cd acts with a broad mechanism of action or with a tissue/organ-specific response, we analyzed the deregulated genes (DEGs) and pathways in the three cell models: hepatic (HepG2), pulmonary (A549) and neuronal (SH-SY5Y) cells. Further, we have investigated the common genes deregulated in all the three cell models to identify any Cd non-specific effects.

### 2.1. Deregulated Pathways in Pulmonary (A549), Hepatic (HepG2) and Neuronal (SH-SY5Y) Cell Models Exposed to Cadmium

**Figure 1:**
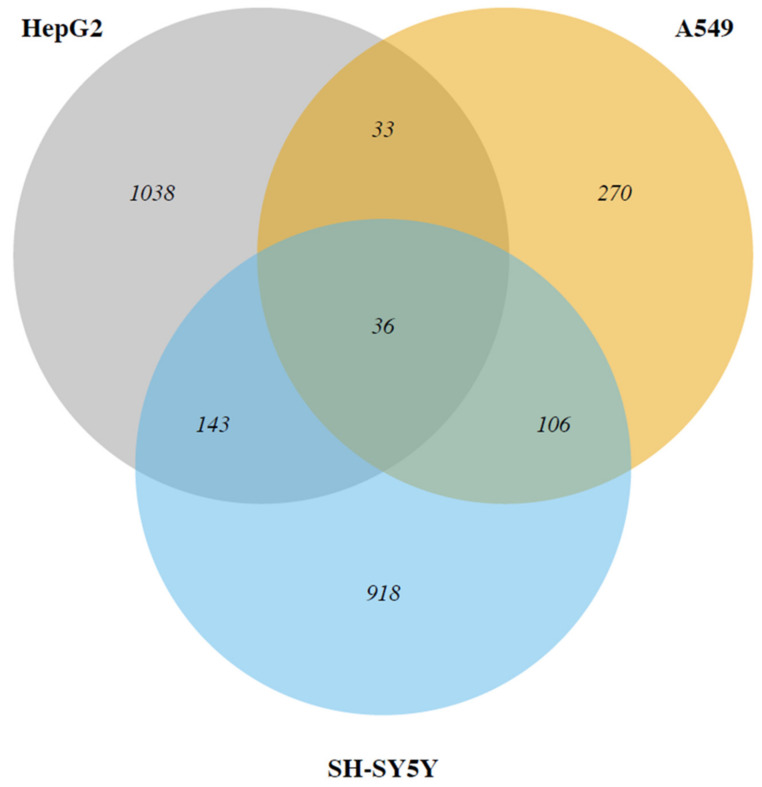
Venn diagram of deregulated genes in hepatic (HepG2), pulmonary (A549), and neuronal (SH-SY5Y) cell models upon cadmium exposure.

The Venn diagram (Figure 1) shows the number of DEGs in the three cell models, and the number of genes in common. As highlighted by the figure, the hepatic cell model displays the highest number of DEGs, followed by the neuronal model and the pulmonary one. A group of only 36 deregulated gene transcripts is in common among the three cell models.

**Figure 2:**
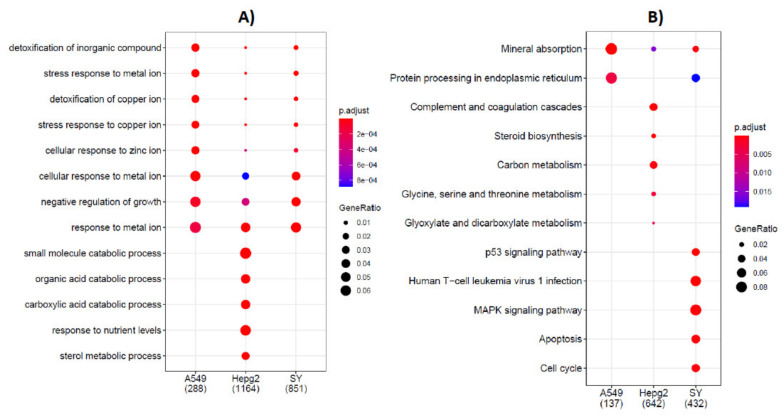
GO term and KEGG pathway enrichment analysis for DEGs after Cd treatment in human pulmonary (A549), hepatic (HepG2) and neuronal (SH-SY5Y) cells. (**A**) GO enrichment analysis. (**B**) KEGG pathway enrichment analysis. The number of DEGs in the three cell models is indicated in parenthesis. ClusterProfiler package was used.

To identify the biological functions of DEGs in the three cell models upon Cd exposure, the genes were subjected to analysis by ClusterProfiler package. The Kyoto Encyclopedia of Genes and Genomes (KEGG) database and gene ontology (GO) were used to run enrichment analysis of DEGs (Figure 2). These analyses provide insights and information about affected biological functions and molecular pathways in the three cell models exposed to cadmium.

The results of GO analysis of biological process are presented in Figure 2A.

HepG2 cells show the highest number of enriched genes (1164), in comparison to SH-SY5Y (851) and A549 (288) cells. GO terms related to metabolic processes were found to be significantly enriched only in HepG2 cells. In particular, catabolic processes of small molecule, organic acid, and carboxilic acid, along with the response to nutrient levels and sterol metabolic processes were found to be significantly enriched in the hepatic cells (Figure 2A, HepG2).

In addition, our data show a number of GO terms in common among the three cell models and related to metal ion(s) cellular and stress response, and detoxification biological processes. The negative regulation of growth is another biological process with DEGs significantly enriched in all the cell models.

The results of KEGG pathway enrichment analysis are displayed in Figure 2B.

Complement and coagulation cascades, steroid biosynthesis, carbon metabolism, amino acids (glycine, serine, and threonine), glyoxylate and dicarboxylate metabolism were significantly enriched in Cd-treated HepG2 cells (Figure 2B, HepG2).

The p53 signaling pathway, human T-cell leukemia virus 1 infection, MAPK signaling pathway, apoptosis and cell cycle were the pathways significantly perturbed by Cd treatment in the neuronal cell model, all pathways with a high percentage of total DEGs in the given term (Gene Ratio, Figure 2B, SH-SY5Y).

The KEGG analysis in pulmonary cell model (Figure 2B, A549) reveals that only 2 pathways with a high gene ratio were perturbed by Cd exposure: protein processes in endoplasmic reticulum and mineral absorption. The mineral absorption, found in hepatic and neuronal cells too, is the unique KEEG pathway enriched in common in the three cell models.

### 2.2. Cadmium Regulates a Group of Genes in Common in HepG2, A549 and SH-SY5Y

A group of 36 regulated gene transcripts is in common among the three cell models exposed to Cd, as evidenced by the Venn diagram (Figure 1).

The list of deregulated (up and down) genes in common in the three cell models is presented in Table 1.

**Figure 3:**
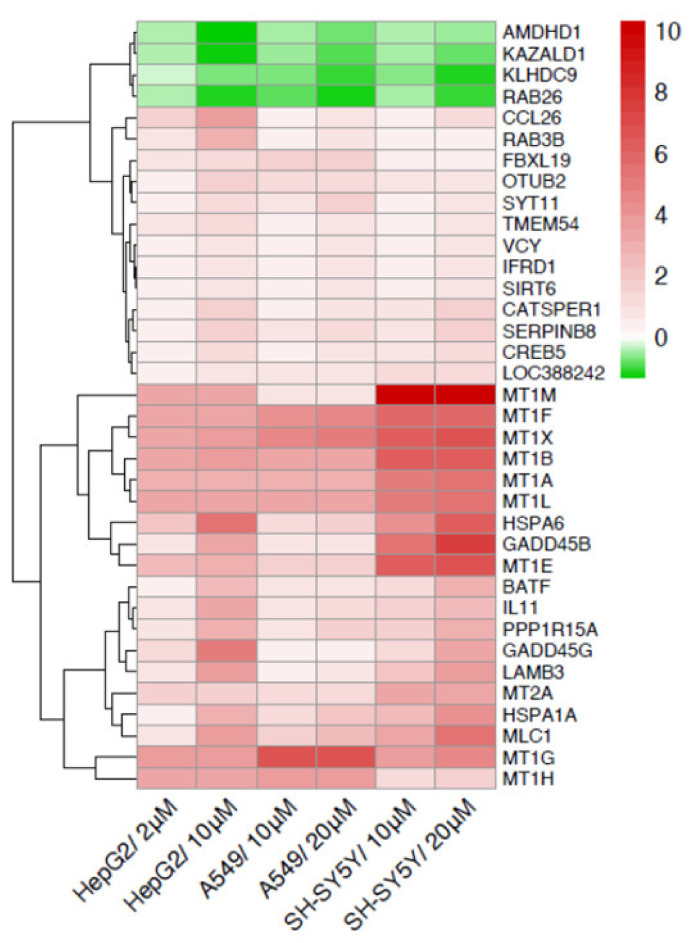
Toxicogenomics of regulated genes in common in the three cell lines. Comparison in human hepatoma cells (HepG2), human lung cancer cells (A549) and human neuronal cells (SH-SY5Y). Heatmap of differentially expressed genes in common in the three cell lines, at the indicated Cd concentrations. The genes name is indicated on the right of the heatmap. Red is used for upregulated genes and green for downregulated ones.

The 36 in common transcripts follow the same pattern of expression, i.e., are upregulated or downregulated in the three cell models analyzed, as shown by the heatmap of Figure 3.

Only 4 out of the 36 genes in common are downregulated: *AMDHD1*, *ZAZALD1, KLHDC9,* and *RAB26.*

The 32 upregulated genes found in common in hepatic, pulmonary end neuronal cells display targets, related to different functions. It is noteworthy that among all upregulated genes, 10 out of 32 represent members of the metallothioneins (*MT*), and 2 genes are members of the heat shock proteins family (*HSPA1A, HSPA6),* responding to metal and proteotoxic stresses. As shown by the heatmap, all *MT* gene members are highly upregulated in the three cell lines.

In Supplementary Table S1 the average expression levels, fold changes and *p*-values of all deregulated genes are presented.

### 2.3. The Two by Two Comparison in the Different Cell Models Reveals Specific Patterns of Expression

**Figure 4:**
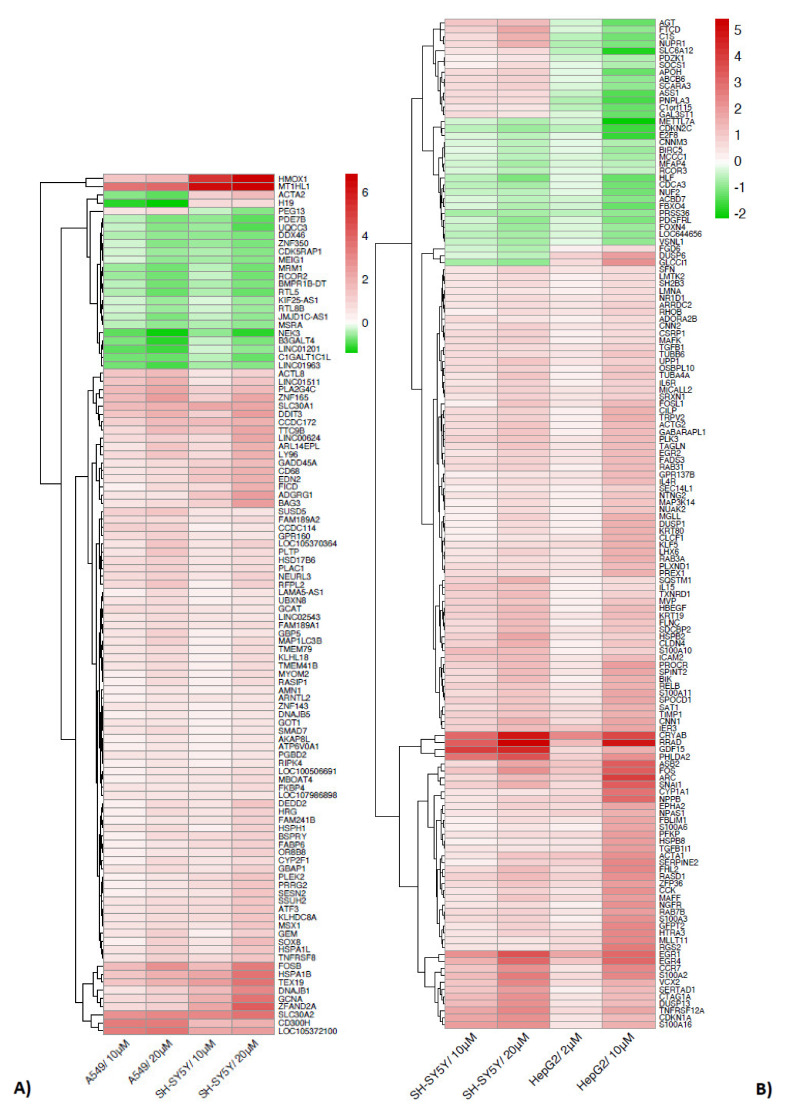

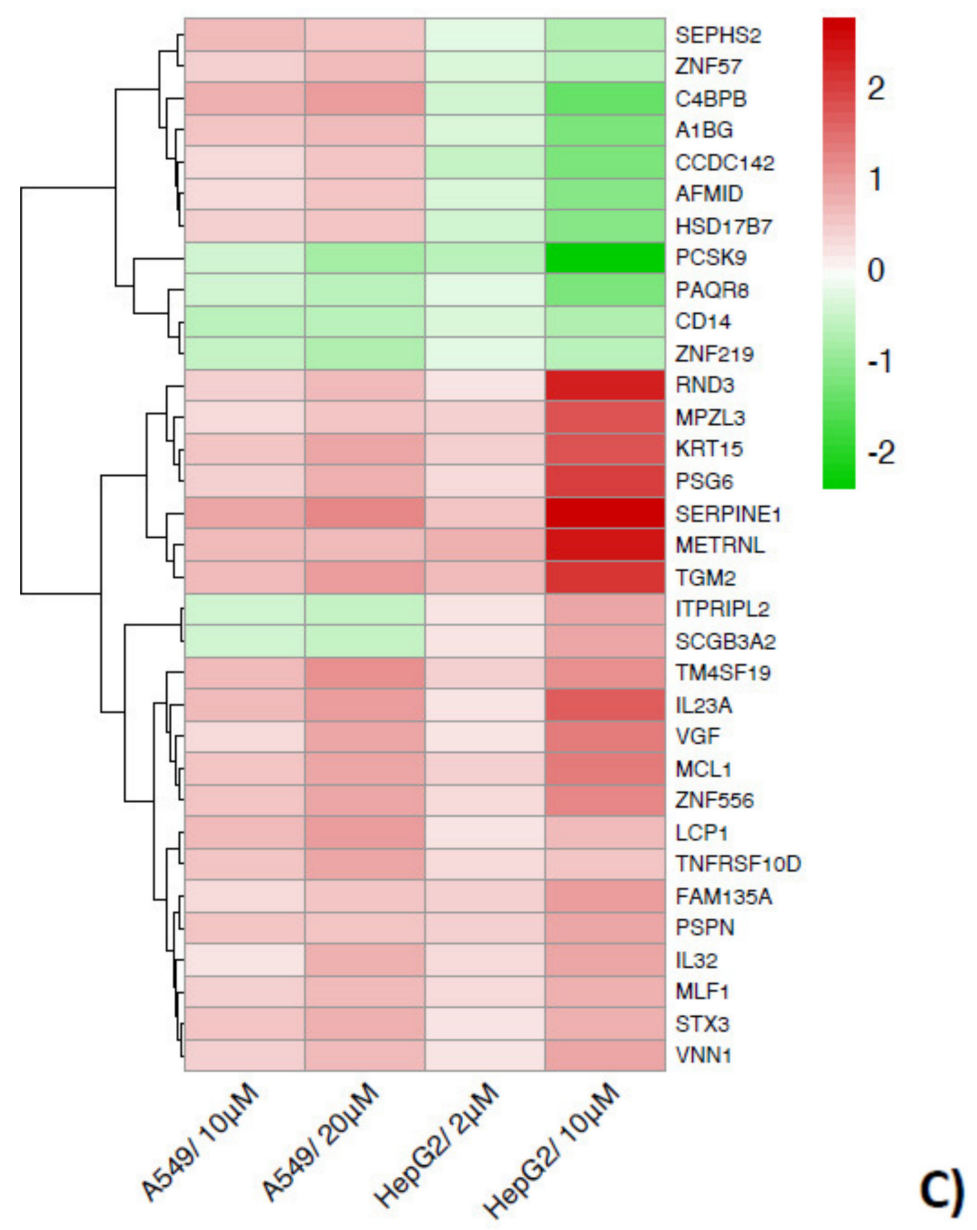
Heatmaps of two by two comparison of regulated genes in human lung cancer cells (A549), human neuronal cells (SH-SY5Y), and human hepatoma cells (HepG2). The cell model used for comparison and Cd concentrations are indicated. (**A**) comparison between A549 and SH-SY5Y cells, (**B**) between SH-SY5Y and HepG2 cells, and in (**C**) A549 and HepG2 are compared. Upregulated (red) and downregulated (green) genes are indicated in the heatmap along with the name of regulated genes.

When comparing two by two the gene transcripts in the cell models used, differences in the pattern of expression (up or down) are evidenced and shown by the heatmaps of Figure 4A–C. The gene transcripts are not individually analyzed, but all the biological functions and pathways of differentially expressed genes (DEGs) in which those genes are selectively and specifically involved are better highlighted in Section 2.1. Some general observations can be made, as follows.

A concentration-response to Cd is observed in some transcripts, both up and downregulated (Figure 4A–C, and Tables S2–S4). Further, the cell models may express different fold changes in response to the same Cd concentrations. For example, A549 and SH-SY5Y cells both upregulate HMOX1 (heme oxygenase 1), but in the neuronal cells the mean fold change (logFC) is 6 in comparison to a mean of 1.5 in pulmonary cells (Figure 4A, Table S2).

In addition, the number of genes regulated with distinct patterns is different when comparing two by two the cell models. A549 and SH-SY5Y have in common the highest number of genes regulated with the same pattern (Figure 4A). Only three genes are regulated with a different pattern: ACTA2 (actin alpha 2, smooth muscle), H19 (H19 imprinted maternally expressed transcript), and PEG13 (paternally expressed 13).

When comparing the pulmonary (A549) and the hepatic (HepG2) cells exposed to the same Cd concentration (10 µM), it is interesting to note that HepG2 cells respond by a general increase (up) and decrease (down) expression level of the same gene transcript.

*ZNF57*, coding for a zinc finger protein, is another example of gene regulated with different pattern in A549 and HepG2 cells.

To highlight at a glance, we have grouped together (Table 2) all genes with different pathway of regulation present in the heatmaps of Figure 4A–C. Note that the colors in the table are not representative of the level of upregulation (red) or downregulation (green); for this detail, refer to the heatmaps of Figure 4, and to the Tables in Supplementary Materials (Tables S2–S4).

### 2.4. Metallothioneins and Heat Shock Proteins Are the Forefront Defense and Response against Cadmium

Metallothioneins (MTs) belong to a group of 15 different families of small-cysteine rich proteins. They are recognized as fundamental in metal ions chelation with primary biological functions of homeostasis of essential trace metals Zn and Cu, and sequestration and protection from environmental toxic elements, such as Cd. Additionally, MTs play essential roles against oxidative stress induced by reactive oxygen and nitrogen species and other free radicals [11].

Hsp70 is one of the members of the heat shock proteins family. This protein plays multiple roles in all stages of protein life from synthesis to degradation, under physiological and stress-induced pathological conditions [12]. Hsp70 is a chaperone acting as a sentinel watching over the cells from deleterious effects caused by a wide range of proteotoxic stresses [13]. The increased expression of this molecular chaperone was previously observed by our group in HepG2 cells under Cd treatment [14].

**Figure 5:**
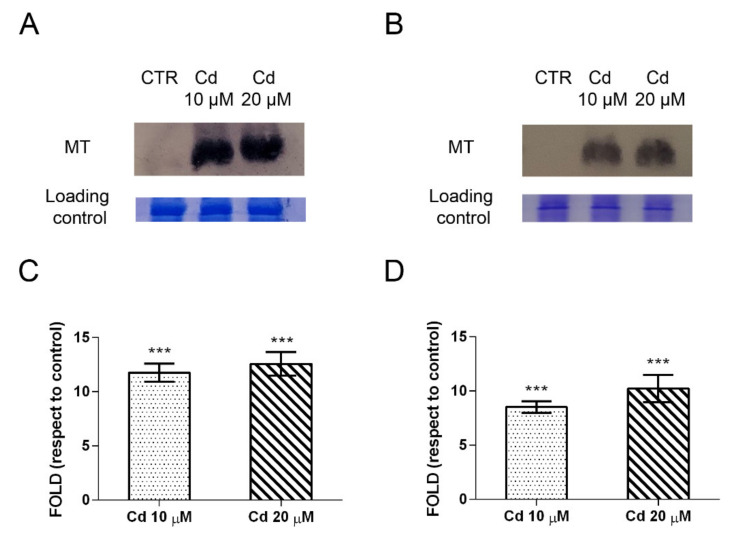
Metallothioneins expression in human lung cancer cells (A549) and human neuronal cells (SH-SY5Y) exposed to cadmium. Representative Western blot performed on A549 (**A**) and SH-SY5Y (**B**) samples exposed to 10 μM and 20 μM Cd for 24 h. Densitometric analyses performed with Scion Image Software are in the panels (**C**,**D**). Values are presented as means ± standard error (SE) of three different experiments. Significantly different from control: *** *p* < 0.001 (Dunnett’s test).

Our immunochemical results show that MTs are highly expressed in human lung (A549) and neuronal (SH-SY5Y) cells exposed to 10 and 20 µM Cd concentrations (Figure 5A,B). High levels of MTs proteins expression are reached at the lowest Cd concentration used, and do not increase dose-dependently (Figure 5C,D). Controls (CTR, Figure 5A,B) show undetectable constitutive levels.

MTs protein induction confirms transcriptomics data in which 10 out of 36 in common genes among the three cell lines are represented by different family members of these metal-binding proteins upregulated by Cd exposure.

Another highly expressed marker is represented by a member of the heat shock proteins family, Hsp70.

**Figure 6:**
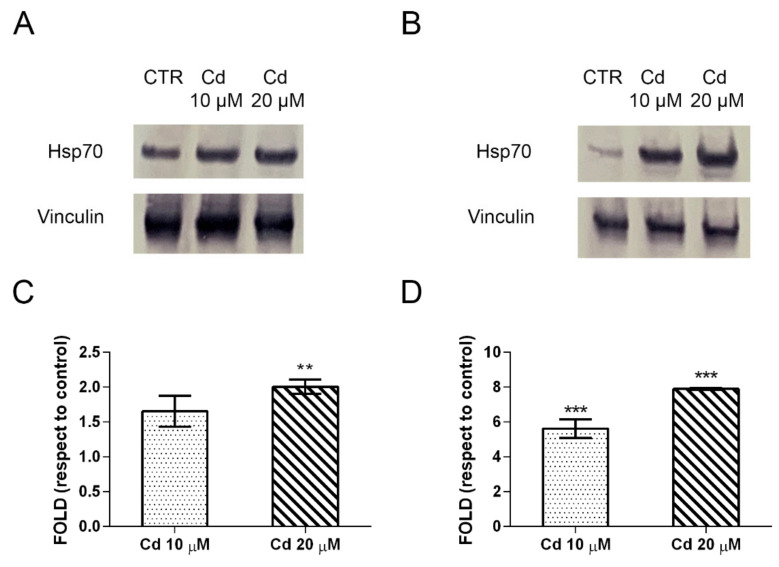
Heat shock protein 70 expression in cadmium-treated A549 and SH-SY5Y cells. Representative Western blots on A549 (**A**) and SH-SY5Y (**B**) samples exposed to 10 μM and 20 μM Cd for 24 h. Densitometric analyses performed with Scion Image Software are in the panels (**C**) (A549 cells) and (**D**) (SH-SY5Y cells). Values are presented as means ± standard error (SE) of three different experiments. Significantly different from control: ** *p* < 0.01; *** *p* < 0.001 (Dunnett’s test).

A549 and SH-SY5Y cells express high levels of this protein upon Cd exposure, as visualized by immunochemical results (Figure 6A,B). An eight-fold higher induction is seen in neuronal cells than in pulmonary cells (Figure 6C,D). The increase of Hsp70 expression level is dose-dependent in SH-SY5Y cells (Cd 10 μM vs. Cd 20 μM, *p* < 0.001).

**Figure 7:**
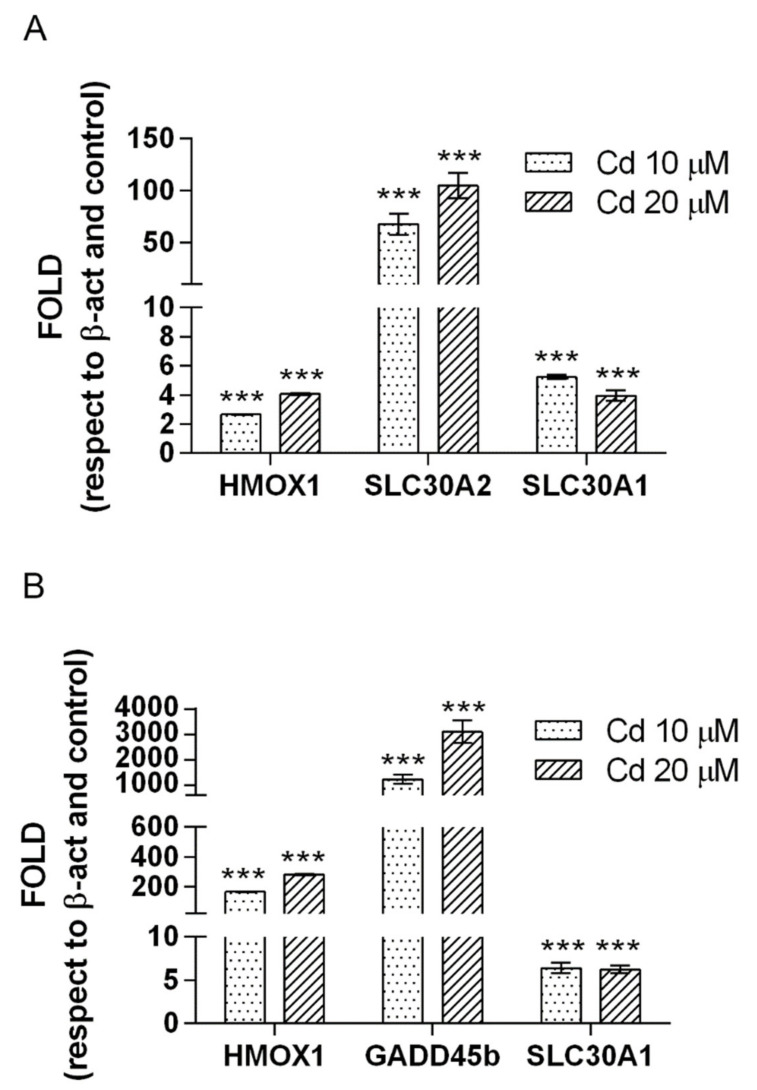
Relative quantification of HMOX1, GADD45β, SLC30A1 and SLC30A2 mRNA levels by real time quantitative PCR. The relative expression levels in A549 (**A**) and SH-SY5Y (**B**) cells exposed to 10 μM and 20 μM Cd for 24 h were expressed as a fold change ± standard error (SE), using β-ACT gene as internal reference, and cells not treated with cadmium as calibrator. Values are presented as means ± standard error (SE) of three different experiments. Significantly different from control: *** *p* < 0.001 (Dunnett’s test).

The immunochemical results on protein expression along with the qRT-PCR results (Figure 7) support and validate the transcriptomics data. Along with HMOX1 and GADD45β, SLC30A1 and SLC30A2 genes were analyzed. These genes, coding for solute carrier family 30 (SLC30A) proteins responsible for cytoplasmic zinc balance by exporting zinc out to the extracellular space or by sequestering cytoplasmic zinc into intracellular compartments, were analyzed due to their role in metal homeostasis regulation and for the results obtained by transcriptomics (see Table S2). An increased expression of HMOX1 and SLC30A2 and SLC30A1 genes transcripts is observed in pulmonary cells (Figure 7A), and increased mRNA transcripts of HMOX1, GADD45β and SLC30A1 in neuronal cells (Figure 7B). The increase of mRNA level is dose-dependent for HMOX, GADD45β and SLC30A2 genes (Cd 10 μM vs. Cd 20 μM, *p* < 0.001).

## 3. Discussion

According to the large number of published papers, Cd causes a multitude of harmful modifications in several tissues-organs, leading to different diseases (e.g., cancer, liver-related diseases, heart and vascular diseases, neurodegeneration). The reported cellular and molecular altered functions in different target tissues (see, e.g., [9,15,16]) frequently include common features, leading to the diverse range of diseases. This aspect arises the question whether any common features and molecular signatures of Cd-induced toxicity and modifications might be the building blocks in initiating and/or contributing to address the cells towards different pathological conditions.

Cd has a similar ionic radius to that of calcium (Ca), and similar electronegativity to that of zinc (Zn) leading to the uptake of this non-essential metal by the same transporters and pathways used by essential elements such as Ca, Zn, iron (Fe) and manganese (Mn) [17]. Within mammalian cells, this molecular mimicry and the chemical properties lead to the substitution for Ca in cellular signaling, the displacement of Zn in the zinc-proteome, the binding to essential sites of biomolecules (e.g., SH-groups) thus impacting the levels of second messengers and transcription factors, inhibiting DNA repair systems and inactivating tumor suppressors such as p53 [16,18,19,20,21].

The ascertained metal elements considered essential for humans include among others, K, Ca, Fe, Cu, and Zn, which participate as cofactors in enzymatic activities and have structural and signaling biological functions.

Further, their concentrations are strictly regulated by specific physiological levels [22]. We have previously reported the interference of Cd with essential metals such as zinc [16,23,24] in processes related to hepatotoxicity, neurotoxicity and carcinogenicity using in vitro models. The Cd-Zn exchange, and Cd effect on Ca signaling pathways and their resultant toxic effects are described in different mammalian cells [25,26]. The interference of Cd with essential elements (Mg, Ca, Fe, Zn, Cu) is reported also in animal models [27].

Applying transcriptomics to three different cell lines (A549, HepG2, and SH-SY5Y cells, respectively) exposed to Cd and representing three major human targets, along with immunochemical analyses, we have identified specificities and commonalities in gene expression.

GO enrichment analysis of DEGs evidenced that the biological functions perturbed in the three cell models had both cell-specific and -common features.

The hepatic cell model showed the highest number of DEGs (1164), as expected from one of the major target organs affected by Cd exposure. In vivo the liver is reported to play an active role in removing Cd ions from blood, thus representing an important risk factor of hepatotoxicity [14]. This leads to Cd accumulation in this organ, and to alteration of metabolic processes. As previously evidenced by our group [28], and shown in this work by GO analysis of DEGs, lipid metabolism and catabolism of small and organic molecules are among the major groups of significantly deregulated processes in the hepatic cell model (HepG2). Other processes significantly deregulated in HepG2 cells evidenced by GO analysis are related to cellular and stress response, and detoxification of inorganic compounds and metal ions (Cu and Zn). Very interestingly, these processes are in common with the other two cell targets analyzed, the pulmonary (A549) and neuronal (SH-SY5Y) cell models and are the unique deregulated processes along with the negative regulation of growth perturbed in the three cell models.

The analysis of DEGs in common in the three cell models helped to have better insights into Cd specific and non-specific mechanisms of toxicity, possibly leading to different pathological conditions in different target and non-target organs.

Despite differences of regulated genes in terms of number and type in the three cell models, only a limited number (36) of dysregulated genes is found in common in the three cell lines, genes displaying the same pattern of expression (up or downregulation).

Only 4 genes were downregulated in all the cell models exposed to Cd. *AMDHD1,* the gene product is a protein which uses Zn and Fe as co-factors and has recently been suggested to play a protective role against breast cancer risk, and it is correlated to Vitamin D levels [29,30]. The gene product of *KAZALD1* is identified as a serum biomarker in gastric cancer, and is found hypermethylated in malignant pleural mesothelioma, while low levels of DNA methylation are found associated with longer survival [31,32]. Another downregulated gene transcript in common in all the three cell models used is *KLHDC9.* Hypermethylation and low gene expression of this gene are suggested to be related to poor prognosis in patients with lung adenocarcinoma [33]. Finally, *RAB26*, the last downregulated gene significantly promotes the migration and invasion of breast cancer cells in Rab26 knockdown sample, and the protein products are important regulators of vesicular fusion and trafficking [34].

Cd ability to induce oxidative stress and generate reactive oxygen species (ROS) is known (see, e.g., [35]), and one of the primary responses of the cell systems is the induction of metallothioneins (MT) and Hsp70 to counteract the oxidative stress and the consequent proteotoxicity. Our results in all the three cell models used are well in agreement with these responses. Transcriptomics and immunochemical results evidence high levels of expression of MT and Hsp70, confirming results formerly published on HepG2 cells [36]. The mechanism accounting for elevated ROS levels upon Cd exposure has been demonstrated by our group in neuronal models. Cd-induced ROS production is caused by a reduction of SOD1 activity due to the substitution of Zn with Cd in the catalytic site of this antioxidant enzyme [37], because of the molecular mimicry of this toxic metal. These previous results well support and explain the current findings of perturbed processes related to ions and Cu and Zn stress and detoxification responses evidenced by GO analyses in all cell models exposed to Cd.

Along with ROS production upon Cd exposure, alteration of Ca signaling is reported (reviewed in [18,35]). This second cell response is probably caused by the interplay of Cd and Ca and the dysregulation of Ca homeostasis [26]. The dysregulation of this second messenger mediates the alteration of mitochondrial morphology and distribution, processes related to neurotoxicity and carcinogenicity, as very recently evidenced by the literature and by our group [25,38].

Strictly related to the stress response to metals, is the group of genes regulated by ions or which represent ions regulators. Apart from the family of *MT*, which receives a special attention further on in this section, there are genes related again to Ca functions (*CATSPER1*), and one gene (*MLC1*) which displays homology with voltage-gated K channel Kv1.1. Both these functions can account again for Cd chemical properties and ability to interfere with and to mimic other metal ions.

Typical genes and oncogenes involved, e.g., in promotion, proliferation, invasiveness, and metastasis (e.g., *BATAF, CREB5, LAMB3*), which accounts for Cd classification as a group I human carcinogen by the International Agency for Research on Cancer (IARC) were found upregulated in all the three cell models analyzed. One of these genes (*OTUB2*) promotes tumorigenesis in non-small cell lung cancer through pathways involving the Warburg effect [39]. The same mechanism affecting the rearrangement of the energy metabolism and exacerbation of the Warburg effect was found in Cd-induced neurotoxicity by our group [37]. A special attention must be given to *GADD45B.* This is described as a predictive biomarker in colon cancer [40], but also for its stress sensors functions in which Zn and dopamine regulate its expression in neuronal cells. Finally, a new role for *GADD45B* in the pathogenesis of Parkinson’s disease is provided by studies both in vivo and in vitro [41]. Additionally, free Zn deficiency induces changes in methylation-related expression of this gene [42].

Another group of interesting genes upregulated in all the three cell models analyzed, are those with immunoregulatory functions and involved in inflammatory process, to which belong chemokines and cytokines (e.g., *CCL26, IL11*). Both immunoregulatory and inflammatory processes are a common characteristic of neurological disorders and cancer.

Cd is regarded as an epigenetic modulator in mammalian cells both in vivo and in vitro, altering the global DNA methylation pattern turning into upregulation of cell protooncogenes and silencing oncosuppressors [35]. It has been reported that short-term Cd exposure reduces DNA methylation, while chronic exposure can result in hypermethylation due to DNA methyltransferase I induction. This discrepancy between short- and long-term Cd exposure on global DNA methylation is not understood, although this change in chromatin methylation may activate signaling pathways responsible for different processes [19]. On this regard and in agreement with literature data, we have found differentially expressed genes such as histone deacetylase or decoding for proteins required in histone acetylation, or proteins expressed upon methylation or acetylation changes. An interesting example is *LAMB3*, which is overexpressed in colorectal cancer (CRC) and is correlated to tumor metastasis and poor prognosis. The dysregulation of LAMB3 in CRC reveals a transcriptional regulation via an acetylation-dependent mechanism [43]. In addition, other genes found dysregulated in our cell models (in common in A549 and SH-SY5Y cells) are *H19* and *PEG13*, both imprinted genes whose product is a long-noncoding RNA, display differences in methylation with dysregulated patterns of imprinting in several cancers [44,45]. Again, related to imprinting, the process that causes genes to be expressed according to their parental origin, *ZNF57* is a gene coding for a zinc finger protein that in eutherians binds specifically to methylated imprinting control regions, and which is regulated with different patterns in our A549 and HepG2 cell.

MiRNAs, small non-coding molecules involved in post-transcriptional regulation of protein expression, are regarded as another epigenetic effect of Cd exposure [9]. We have previously demonstrated [28] the downregulation of miRNAs described as tumor suppressors and involved in cancer-related pathways in HepG2 cells exposed to Cd. Interestingly, in this work, Cd is found to upregulate *TMEM54*, which is described with regulatory functions on miRNAs expression by in silico studies and involved in the development of nasopharyngeal carcinoma [46].

Regarding Cd-induced neuronal damage and the possible mechanisms involved, the oxidative stress, interference with Ca, and Zn-dependent processes and apoptosis induction are the main processes reported [47]. In addition, unbalanced and dysregulated levels of essential metals, such as Zn, Cu, Mn and Fe, participate in the activation of the inflammatory signaling pathway, impair structural, regulatory and catalytic functions of many enzymes, receptors and transporters, and have a role in the pathogenesis of neurodegenerative disorders in exposed population, as recently demonstrated by our group [48]. Neurodegeneration occurs through the association of dysregulated metals with proteins and consequent formation of aggregates (e.g., fibrillar β-amyloid aggregation), as well described in [49].

Special attention has to be given to the family members of *MT* genes, coding for small cysteine-rich proteins, the family of metallothioneins, which represent the common denominator and the common feature in the three cell models used. The best-known biological function of these proteins is the binding of metal ions in metal homeostasis processes and protection against heavy metal toxicity. *MTs* are genes upregulated by a metal-induced stress (including oxidative stress), which is a well-known function. However, new insights into MT roles in oncogenesis, carcinogenesis and chemoresistance are emerging and reviewed in [50]. In addition, the multifunctionality properties of MT are also explained by their enhanced expression by inflammatory stimuli and conditions [51], and their modulation by epigenetic modifications in MT promoter regions [11].

The multifunctional role of MT, and the involvement of metal homeostasis in different processes that emerges from our transcriptomics data is well described and summarized by the functional analysis of single genes dysregulated (up or down) in common in the pulmonary, hepatic, and neuronal human cell models which unveil interesting insights. Our results show that most biological processes enriched in the cell models analyzed are related to cellular responses to metal ion(s) (zinc and copper). In addition, the KEGG pathways’ analysis in differentially expressed genes (DEGs) shows for the first time that the only pathway dysregulated in common in the three cell models is the mineral absorption pathway. These results strengthen the connection between interference of Cd with essential elements, and the development of different diseases.

Overall, a general picture emerges from our transcriptomics and immunochemical data in the three cell models used, hepatic, pulmonary and neuronal. Our data, along with those of the literature, clearly describe how the molecular mimicry of Cd and its ability to cause a general metal ions dyshomeostasis represent the initial common key event leading to different molecular signatures and alterations later on. An additional molecular signature that emerges from our study is the epigenetic role of Cd combined to its dyshomeostatic, deconstructing and inactivating properties. These features are ultimately responsible for different diseases, no matter the tissue (cell model) affected. Thus, the maintenance of a good metal balance to counteract Cd exposure seems to be the hub in the complex cellular and molecular interaction networks.

Future studies should address closely the control of Cd uptake and the restoring of the physiological balance of essential metals. The re-establishment of metal homeostasis is of keen importance for all signaling and regulatory processes and it has structural and catalytic roles, as for example in the zinc-proteome which accounts for around 3000 proteins and enzymes.

## 4. Materials and Methods

### 4.1. Cell Culture and Treatment

We have analyzed and compared transcriptomics data from human hepatoma cells (HepG2), human lung carcinoma cells (A549), and human neuroblastoma cells (SH-SY5Y) exposed to CdCl_2_ (Cd) for 24 h. The three cell lines are used as a model for hepatic, lung, and neuronal cells targets, and were originally obtained by the American Type Culture Collection (HepG2 ATCC^®^ HB-8065™, A549 ATCC^®^ CCl-185™, and SH-SY5Y ATCC^®^ CRL-2266™). Previous transcriptomics data from HepG2 cells [28] were used and reanalyzed for comparison with new data obtained from A549 and SH-SY5Y cells. Cd concentrations were selected on the basis of cytotoxicity data. Namely, two non-cytotoxic concentrations were used in each cell model to evidence any possible concentration-responses, and one of the used concentrations is in common among the three cell lines. Cytotoxicity was determined spectrophotometrically by reduction of MTT (metabolic assay) (Sigma Chemical Co., St. Louis, MO, USA) by the mitochondrial dehydrogenase of viable cells to a blue formazan product [15] (see Figure S1 and [38]). The cells were seeded in 96-well plates at a density of 1 × 10^4^ cells/well and after 24 h were treated with different CdCl_2_ concentrations (0–100 µM). After 24 h the medium was replaced with a complete medium without phenol red, and 10 µL of 5 mg/mL MTT solution was added to each well. After a further 4 h incubation time at 37 °C, absorbance was measured at 570 nm using a microplate reader upon solubilization of formazan salts by 10% Triton X-100, 0.1 N HCl in isopropanol. Viabilities were expressed as a percentage of the control. Nonlinear regression of experimental data was obtained using a four-parameter logistic curve (SigmaPlot 9.0 software).

A549 cells were cultured in Opti-MEM medium (Gibco, Thermo Fisher, Monza, Italy) supplemented with 10% fetal bovine serum, and 1% antibiotics at 37 °C under a humified atmosphere of 5% CO_2_ in air. SH-SY5Y cells were cultured in Eagle Minimum Essential Medium (EMEM) and F12 medium (1:1) supplemented with 10% fetal bovine serum and 1% antibiotics under the same atmospheric conditions.

The cells (A549 and SH-SY5Y) were seeded either in 100 mm Ø dish for RNA extraction and transcriptomics analysis at 1 × 10^6^ cells/dish, or in 162 cm^2^ growth area flask at 3 × 10^6^ cells/flask for biochemical analyses. All treatments were performed 24 h after cell seeding at 10 and 20 µM Cd diluted in complete culture medium and the cells were incubated for 24 h. Neuronal and pulmonary cells cultured in complete medium were used as controls.

### 4.2. Microarray Expression Profiling

Total RNA purification from A549 and SH-SY5Y cells, and the microarray experiments were essentially performed according to [16]. Briefly, immediately at the end of treatments, RNA was purified using the RNeasy Plus kit (Qiagen, Milan, Italy), quantified using an ND-1000 UV–vis spectrophotometer (Thermo Scientific, Waltham, MA, USA) and the integrity assessed with the Agilent 2100 Bioanalyzer (Agilent Technologies Inc., Santa Clara, CA, USA), according to the manufacturer’s instructions. All RNA samples used had a 260/280 ratio above 1.9 and an RNA Integrity Number above 9.0.

The microarray experiment comprised 2 biological replicates for the controls, and 3 biological replicates for the treated cells, both pulmonary and neuronal.

All details of microarray experiments on HepG2 cells are found in [28]. Pulmonary and neuronal cell models were processed according to previously described protocols [28]. All sample-labelling, hybridization, washing and scanning steps were performed according to manufacturer’s instructions. Briefly, Cy3-labeled cRNA was generated from 500 ng input total RNA using Quick Amp Labeling Kit, One-color (Agilent). The slices were then scanned with the Agilent G2565BA Microarray Scanner (Agilent).

All microarray data are deposited and available at NCBI’s Gene Expression Omnibus repository [52] under accession numbers GSE31286 (HepG2 cells), GSE190100 (A549 cells), and GSE190101 (SH-SY5Y cells).

The Agilent microarray data were pre-processed in a one-color channel mode. The green color was collected, and the background intensity of each spot was subtracted from the foreground intensity using the normexp method and with an offset of 20. A quantile normalization was performed for normalization between arrays and signals for replicated spots were averaged.

Multidimensional scaling (MDS) was performed after filtering probes that were not detected or detected at low level. A normalized log2 expression of at least 6 in at least 2 samples was used for the expression filtering. The statistics were performed using the R environment (version 4.1.1). The limma test (implemented in limma version 3.46.0) was also conducted on the filtered dataset, comparing cadmium treated samples versus non treated control samples. A false discovery rate (FDR) lower than 0.05 in at least one of the Cd concentrations used, combined with an absolute log2 fold change of 0.20 for the lowest concentration and 0.5 for the highest concentration, was used to obtain the differentially expressed genes (DEGs). The heatmaps were made using the pheatmap library (version 1.0.12).

The clusterProfiler package was used to annotate and enrich the gene ontology (GO, Biological process) and Kyoto Encyclopedia of Genes and Genomes (KEGG) pathways for differentially expressed genes (DEGs). Function annotation of DEGs is helpful to find out the biological functions and pathways of the regulated genes by cadmium in the three cell lines. *p* < 0.05 was used as a cut-off criterion for significant enrichment [53].

### 4.3. Immunochemical Analysis of Metallothioneins and Heat Shock Proteins

A549 pulmonary cells and SH-SY5Y neuronal cells were treated for 24 h at 10 and 20 µM Cd concentrations for 24 h. At the end of treatment period, the cells were differentially processed to obtain cell extracts for low molecular weight (metallothioneins, MTs) and for heat shock 70 protein (hsp70) expression analysis.

#### 4.3.1. Metallothioneins Extraction and Expression

The cells were essentially processed according to [16] to obtain cell lysate of low molecular weight proteins. Briefly, the cells were harvested, cold-PBS washed and centrifuged. The cell pellets were resuspended in 10 mM Tris-HCl buffer (pH 7) supplemented with 5 mM EDTA and 1mM phenylmethylsulfonyl fluoride as a protease inhibitor. The samples were immediately frozen (−20 °C) to allow membranes breaking. Defrosted samples (lysate) were clarified by high-speed centrifugation (20,000× *g*, 45 min) to separate low molecular weight proteins, including the MTs (<7 kDa). An aliquot of clarified samples (supernatants) was used for total protein content [23], the remaining volume was diluted 1:1 in sample buffer (0.25 M Tris-HCl, pH 6.8, 2% SDS, 30% glycerol, 10% β-metrcaptoethanol, 0.01% bromophenol blue) and stored at −20 °C until use.

In this case, 20 µg of total proteins were separated on gradient 4–12% gel (Invitrogen Thermo Fisher Scientific) and transferred to nitrocellulose. Western blotting was performed according to [36] using a mouse anti-metallothionein antibody (Zymed, Thermo Fisher Scientific) that binds MT-I and MT-II isoforms. Gels from the same cell extracts were stained (Coomassie Blue) for visualization of correct cell loading.

Densitometric analysis by Scion Image software (Scion Corp. Fredrick, Walkersville, MD, USA) provided the quantification of proteins expression (MTs) in Cd-treated samples compared to controls.

#### 4.3.2. Heat Shock Proteins Extraction and Expression

All cell samples (control and Cd-treated) were harvested, centrifuged and pellets were obtained by further centrifugation in PBS containing protease inhibitors. Homogenates were obtained by passing the cell samples though a syringe needle (22–23 ga diameter) and by incubation on ice for 15 min. The samples were then sonicated (10–15 s on ice) and centrifuged for the separation of the proteins of interest. The supernatants were stored at −80 °C in sample buffer until use. In this case, 30 µg of proteins were used and separated on 7% gel (Invitrogen Thermo Fisher Scientific) and transferred onto nitrocellulose membrane. A mouse monoclonal anti-Hsp70 antibody (Enzo Life Sciences, Euroclone, Pero-MI, Italy) was used for protein visualization. The same cell extracts were incubated with anti-vinculin antibody (Enzo Life Sciences, Switzerland) for correct protein loading visualization.

Densitometric analysis by Scion Image software (Scion Corp. Fredrick, MD, USA) provided the quantification of proteins expression (hsp70) in Cd-treated samples compared to controls.

### 4.4. Determination of Gene Expression Level by Quantitative Reverse Transcription (qRT-PCR)

SH-SY5Y and A549 cells were seeded at 1 × 10^6^ cells/100 mm dish and after 24 h were treated with 10 or 20 μM CdCl_2_ for 24 h. The total RNA was isolated using Quick-RNATM MiniPrep (Zymo Research, Irvine, CA, USA), according to manufacturer’s instructions. Total RNA was reverse-transcribed using SuperScript II Reverse Transcriptase (Invitrogen, Carlsbad, CA, USA), oligo dT and random primers, according to the manufacturer’s protocol. For quantitative real-time PCR (qPCR), SYBR Green method was used to evaluate growth arrest and DNA damage-inducible protein GADD45β and heme oxygenase 1 expression in SH-SY5Y cells and zinc transporters SLC30A1 and SLC30A2 and heme oxygenase 1 expression in A549 cells. Briefly, 50 ng cDNA was PCR amplified with Luna^®^ Universal qPCR Master Mix (New England BioLabs, Hitchin, Hertfordshire, UK) and specific primers, using an initial denaturation step at 95 °C for 10 min, followed by 40 cycles of 95 °C for 15 sec and 59 °C annealing for 1 min. Each sample was normalized using β-actin gene as an internal reference control. The relative expression level was calculated with the Livak method (2[-ΔΔC(T)]) and was expressed as a relative fold change between Cd treated and untreated cells [24].

The primers used for qPCR are the following: GADD45β Fw 5′-CAGAAGATGCAGACGGTGAC-3′ and Rv 5′-AGGACTGGATGAGCGTGAAG-3′; HMOX1 Fw 5′-TGCCCCAGGATTTGTCAGAG-3′ and Rv 5′-AAGTAGACAGGGGCGAAGAC-3′; SLC30A2 Fw 5′-TGCACCTTCGTCTTCTCCATC-3′ and Rv 5′- GCGATGTGGACAGACAGAAC-3′; SLC30A1 Fw 5′- GCGCTGACCTTCATGTTCAT-3′ and Rv 5′- AGTCAGGAAGATGGCGTTCA-3′; β-ACT Fw 5′-CGACAGGATGCAGAAGGAG-3′ and Rv 5′-ACATCTGCTGGAAGGTGGA-3′.

### 4.5. Statistical Analysis of Immunochemical and qPCR Data

The relative fold changes from qPCR and densitometric data from western blot were tested by Dunnett’s multiple comparison procedure. To evaluate dose-dependent response, Cd 10 μM and Cd 20 μM contrast was tested by Student’s *t*-test. Results were considered statistically significant at *p* < 0.05. All calculations were conducted using the R statistical programming environment (R.C. Team, R: A Language and Environment for Statistical Computing, R Found. Stat. Comput, Vienna, Austria, 2008 https://www.r-project.org/ last accessed on 2 February 2022). All treated samples were compared to their reference controls.

## Figures and Tables

**Table 1:** List of deregulated genes in common in HepG2, A549 and SH-SY5Y cells.

Gene	Description	Gene	Description
*AMDHD1*	amidohydrolase domain containing 1	*MT1B*	metallothionein 1B
*BATF*	basic leucine zipper ATF-like transcription factor	*MT1E*	metallothionein 1E
*CATSPER1*	cation channel sperm associated 1	*MT1F*	metallothionein 1F
*CCL26*	C-C motif chemokine ligand 26	*MT1G*	metallothionein 1G
*CREB5*	cAMP responsive element binding protein 5	*MT1H*	metallothionein 1H
*FBXL19*	F-box and leucine rich repeat protein 19	*MT1L*	metallothionein 1L, pseudogene
*GADD45B*	growth arrest and DNA damage inducible beta	*MT1M*	metallothionein 1M
*GADD45G*	growth arrest and DNA damage inducible gamma	*MT1X*	metallothionein 1X
*HSPA1A*	heat shock protein family A (Hsp70) member 1A	*MT2A*	metallothionein 2A
*HSPA6*	heat shock protein family A (Hsp70) member 6	*OTUB2*	OTU deubiquitinase, ubiquitin aldehyde binding 2
*IFRD1*	interferon related developmental regulator 1	*PPP1R15A*	protein phosphatase 1 regulatory subunit 15A
*IL11*	interleukin 11	*RAB26*	RAB26, member RAS oncogene family
*KAZALD1*	Kazal type serine peptidase inhibitor domain 1	*RAB3B*	RAB3B, member RAS oncogene family
*KLHDC9*	kelch domain containing 9	*SERPINB8*	serpin family B member 8
*LAMB3*	laminin subunit beta 3	*SIRT6*	sirtuin 6
*LOC388242*	SAGA complex associated factor 29 pseudogene	*SYT11*	synaptotagmin 11
*MLC1*	modulator of VRAC current 1	*TMEM54*	transmembrane protein 54
*MT1A*	metallothionein 1A	*VCY*	variable charge Y-linked

In grey are the downregulated genes. All the details on average expression, log fold change and significance (adjusted *p*-value) are in Supplementary Table S1.

**Table 2:** Genes in common in HepG2, A549 and SH-SY5Y cells displaying different patterns of expression emerged from the two by two comparison. Note that colors are indicative of upregulation (red) or downregulation (green) only, not of the level of expression.

Gene	Description	A549	HepG2	SH-SY5Y
*ACTA2*	actin alpha 2, smooth muscle			
*H19*	H19 imprinted maternally expressed transcript			
*PEG13*	paternally expressed 13			
*SEPHS2*	selenophosphate synthetase 2			
*ZNF57*	zinc finger protein 57			
*C4BPB*	complement component 4 binding protein beta			
*A1BG*	alpha-1-B glycoprotein			
*CCDC142*	coiled-coil domain containing 142			
*AFMID*	arylformamidase			
*HSD17B7*	hydroxysteroid 17-beta dehydrogenase 7			
*ITPRIPL2*	ITPRIP like 2			
*SCGB3A2*	secretoglobin family 3A member 2			
*AGT*	angiotensinogen			
*FTCD*	formimidoyltransferase cyclodeaminase			
*C1S*	complement C1s			
*NUPR1*	nuclear protein 1, transcriptional regulator			
*SLC6A12*	solute carrier family 6 member 12			
*PDZK1*	PDZ domain containing 1			
*SOCS1*	suppressor of cytokine signaling 1			
*APOH*	apolipoprotein H			
*ABCB6*	ATP binding cassette subfamily B member 6 (Langereis blood group)			
*SCARA3*	scavenger receptor class A member 3			
*ASS1*	argininosuccinate synthase 1			
*PNPLA3*	patatin like phospholipase domain containing 3			
*C1orf115*	chromosome 1 open reading frame 115			
*GAL3ST1*	galactose-3-O-sulfotransferase 1			
*FGD6*	FYVE, RhoGEF and PH domain containing 6			
*DUSP6*	dual specificity phosphatase 6			
*GLCCI1*	glucocorticoid induced 1			

## Data Availability

Microarray data of HepG2 cells are available under accession number GSE31286 of NCBI’s Gene Expression Omnibus repository. A549 and SH-SY5Y data are deposited at the NCBI’s Gene Expression Omnibus repository under accession numbers GSE190100 and GSE190101, respectively.

## Supplementary Materials

The following are available online at https://www.mdpi.com/article/10.3390/ijms23031768/s1.
