# Supplementary material for: Is Cadmium Toxicity Tissue-Specific? Toxicogenomics Studies Reveal Common and Specific Pathways in Pulmonary, Hepatic, and Neuronal Cell Models
Source: Int J Mol Sci. 2022 Feb 4;23(3):1768. doi: 10.3390/ijms23031768 (PMC8836438; doi:10.3390/ijms23031768)
Supplement: Supplementary file 1 [file ijms-23-01768-s001.zip › Table S1.pdf]

Table S1. Regulated genes (up and down) in common in hepatic (HepG2), pulmonary (A549), and neuronal (SH-SY5Y) cell models exposed to Cd for 24 hours.

Gene	AveExpr. A549	AveExpr .SHSY5Y	AveExpr. HepG2	logFC.10 A549	logFC.20 A549	logFC.10 SHSY5Y	logFC.20 SHSY5Y	logFC.2 HepG2	logFC.10 HepG2	adj.P.Val.10 A549	adj.P.Val.20 A549	adj.P.Val.10 SH5Y5Y	adj.P.Val.20 SHSY5Y	adj.P.Val.2 HepG2	adj.P.Val.10 HepG2	Description
AMDHD1	9,2	6,6	9	-0,5	-0,7	-0,4	-0,5	-0,4	-1,3	0,25139456	0,03033218	0,15398746	0,01571098	0,9457244	0,0040933	amidohydrolase domain containing 1
BATF	6,1	7,3	9,9	0,6	0,9	1,2	2,8	0,6	2,5	0,20794761	0,02888374	0,43993755	0,01998279	0,945262	0,00127735	basic leucine zipper ATF-like transcription factor
CATSPER1	7,6	6,2	6,6	0,6	0,8	0,9	1,5	0,6	1,6	0,12146093	0,01398132	0,02635297	0,00096511	0,9997134	0,02377807	cation channel sperm associated 1
CCL26	8,9	5,9	7,1	0,5	0,8	0,6	1,2	1,8	3,7	0,24471985	0,0176621	0,40246302	0,02484106	0,0632238	0,0002156	26
CREB5	5,9	7,1	6,5	0,4	0,7	0,7	1,4	0,3	1,4	0,1834035	0,00838282	0,05297719	0,00096511	0,9904707	0,00119961	cAMP responsive element binding protein 5
FBXL19	11,5	10	10,4	1,6	1,7	0,5	0,6	0,8	1,3	7,71E-05	3,63E-05	0,12168639	0,01621177	0,5732982	0,00413852	F-box and leucine rich repeat protein 19
GADD45B	12,6	10,6	10,1	0,6	0,9	5,5	7,7	0,7	3,3	0,08717041	0,00559998	0,01122615	0,00055863	0,5198574	8,87E-05	growth arrest and DNA damage inducible beta
GADD45G	6,5	10,3	7,5	0,3	0,5	1,2	3,2	1,1	4,9	0,2339901	0,02478063	0,36732925	0,00857652	0,4631329	8,87E-05	growth arrest and DNA damage inducible gamma
HSPA1A	15,5	14,2	13,5	1,1	2	2,4	4,1	0,4	3	0,07657072	0,00145018	0,09556278	0,00324922	0,9997134	0,00069383	heat shock protein family A (Hsp70) member 1A
HSPA6	7,5	9,5	8	1,1	1,6	4,3	6,4	2	5,6	0,00239558	0,00010377	0,01315248	0,00055859	0,109739	9,60E-05	heat shock protein family A (Hsp70) member 6
IFRD1	10,9	10	10,8	0,4	0,7	0,4	0,9	0,3	0,7	0,32942473	0,04290565	0,40752718	0,01421756	0,9120202	0,00636209	interferon related developmental regulator 1
IL11	10,7	8,7	10,1	0,9	1,4	1,6	2,7	0,8	3,2	0,12146093	0,0048584	0,0391443	0,00101022	0,643273	0,00025195	interleukin 11
KAZALD1	9,1	8,2	10,3	-0,5	-0,8	-0,4	-0,8	-0,4	-1,2	0,27784371	0,0249525	0,1712005	0,00628738	0,9854096	0,00789259	Kazal type serine peptidase inhibitor domain 1
KLHDC9	8,4	7,9	9,6	-0,6	-1	-0,6	-1,1	-0,2	-0,7	0,13317157	0,00621907	0,15612226	0,00582294	0,9997134	0,02149352	kelch domain containing 9
LAMB3	11,7	8	9,3	0,4	0,7	2,2	3,7	0,9	4	0,19798177	0,01218963	0,05635801	0,00166856	0,9997134	0,00205931	laminin subunit beta 3
LOC388242	13	8,3	9,4	0,6	0,7	1,2	1,3	0,3	0,8	0,0674357	0,0205932	0,00707905	0,0009668	0,9591153	0,00810221	SAGA complex associated factor 29 pseudogene
MLC1	6,6	8,6	6,7	1,5	2,6	3,5	5,5	0,9	3,7	0,15080498	0,0048584	0,0667328	0,00279908	0,4265658	9,59E-05	1
MT1A	17,3	16,1	16	2,8	2,8	5,1	5,5	3	3,1	4,75E-05	4,05E-05	1,48E-06	5,54E-07	0,0081143	0,00064437	metallothionein 1A
MT1B	16,8	15,3	15,7	3,4	3,5	6,1	6,2	3,5	3,7	6,59E-07	5,05E-07	7,96E-08	3,34E-08	0,0080946	0,00046514	metallothionein 1B
MT1E	9,5	11,7	11,4	1,5	1,5	6,3	6,6	2,4	2,9	0,00017569	0,00011523	1,48E-06	5,54E-07	0,0632238	0,00151072	metallothionein 1E
MT1F	14,9	13,6	13,3	4,3	4,5	5,7	5,9	3,5	3,3	3,45E-06	2,40E-06	1,48E-06	5,54E-07	0,0340246	0,002344	metallothionein 1F
MT1G	10,7	8,4	14,8	6,7	6,8	3,7	4,5	3,7	3,8	9,30E-07	8,73E-07	0,002024	0,00021357	0,0088442	0,00077032	metallothionein 1G
MT1H	7,8	6,2	15,8	3,7	3,8	1,3	1,6	3,5	3,6	2,24E-05	1,56E-05	0,00834876	0,00062208	0,0081143	0,00067589	metallothionein 1H
MT1L	16,4	14,2	15,9	3,3	3,3	5,3	5,6	3,3	3,4	6,67E-07	5,20E-07	1,28E-05	3,35E-06	0,0081143	0,00060896	pseudogene
MT1M	5,8	12,3	14,7	0,7	0,7	9,9	10,3	3,5	3,6	0,03312847	0,0120383	1,48E-06	5,54E-07	0,0299446	0,00154552	metallothionein 1M
MT1X	15,6	14,3	15,8	4,6	4,9	6,4	6,7	3,5	3,7	1,95E-07	1,15E-07	1,26E-08	4,52E-09	0,0080946	0,00048298	metallothionein 1X
MT2A	17,3	15,8	16,6	1,3	1,2	3,5	3,4	1,6	1,8	0,00044933	0,00057602	3,10E-06	1,83E-06	0,0715937	0,00243717	metallothionein 2A
OTUB2	7,3	7,4	7,5	1,1	1,3	0,8	0,8	0,4	1,8	0,00651478	0,00124658	0,00935147	0,00125484	0,5822455	0,00012592	ubiquitin aldehyde binding 2
PPP1R15A	9,7	10	9,3	0,9	1,8	1,6	3,1	0,6	3,1	0,06233089	0,00042734	0,17125605	0,00533278	0,9079092	0,00057019	protein phosphatase 1 regulatory subunit 15A
RAB26	11,5	8,7	7,6	-0,8	-1,2	-0,5	-1	-0,4	-1,1	0,14198852	0,01249787	0,51180842	0,04203412	0,7431268	0,00192278	RAB26, member RAS oncogene family
RAB3B	9,8	7	8,6	0,5	1	0,4	0,6	0,7	2,8	0,20394935	0,00538389	0,09155064	0,00468029	0,6243907	0,00024742	RAB3B, member RAS oncogene family
SERPINB8	7,4	6,4	8,8	0,9	1,1	1	1,6	0,6	1,7	0,023821	0,00407349	0,1851281	0,00866941	0,9457244	0,00641039	serpin family B member 8
SIRT6	9,9	10,1	9	0,4	0,7	0,5	0,9	0,3	0,7	0,28003399	0,01940125	0,21194635	0,01186799	0,9997134	0,04425327	sirtuin 6
SYT11	6,4	10,7	5,9	1	1,6	0,5	0,7	0,2	1,3	0,04566584	0,00098203	0,13316459	0,00935158	0,9997134	0,00261459	synaptotagmin 11
TMEM54	13	12,2	8,1	0,4	0,8	0,5	0,7	0,8	1,4	0,32056332	0,04324383	0,13120964	0,00802322	0,141681	0,00052364	transmembrane protein 54
VCY	7,8	6,5	7,1	0,4	0,8	0,3	0,9	0,3	1	0,36660865	0,03500513	0,50520761	0,01517863	0,8412789	0,00270785	variable charge Y-linked
