# Supplementary material for: Is Cadmium Toxicity Tissue-Specific? Toxicogenomics Studies Reveal Common and Specific Pathways in Pulmonary, Hepatic, and Neuronal Cell Models
Source: Int J Mol Sci. 2022 Feb 4;23(3):1768. doi: 10.3390/ijms23031768 (PMC8836438; doi:10.3390/ijms23031768)
Supplement: Supplementary file 1 [file ijms-23-01768-s001.zip › Table S2.pdf]

Table S2. Comparison of regulated genes (up and down) in common in pulmonary (A549), and neuronal (SH-SY5Y) cell models exposed to Cd for 24 hours.

	AveExpr. A549	AveExpr. SHSY5Y	logFC.10. A549	logFC.20. A549	logFC.10 .SHSY5Y	logFC.20. SHSY5Y	adj.P.Val.10 .A549	adj.P.Val.20. A549	adj.P.Val.10.S HSY5Y	adj.P.Val.20. SHSY5Y	Description
ACTA2	8,6245	7,96338	-0,6403	-0,8322	1,19265	1,533856	0,19327779	0,0327109	0,017505413	0,00127934	actin alpha 2, smooth muscle
ACTL8	8,34331	6,30257	1,316438	1,56929	0,66143	1,099943	0,00636561	0,00095894	0,371753983	0,03907408	actin like 8
ADGRG1	14,1145	8,98864	0,434868	0,51455	1,27846	2,482166	0,14198852	0,02697446	0,163404333	0,0048983	adhesion G protein-coupled receptor G1
AKAP8L	10,8004	10,7869	0,375127	0,59423	0,3186	0,523067	0,27682879	0,03647453	0,167276212	0,00816702	A-kinase anchoring protein 8 like
AMN1	8,11753	6,74308	0,217663	0,609	0,30403	0,613949	0,51093866	0,04290565	0,238487986	0,00816702	antagonist of mitotic exit network 1 homolog
ARL14EPL	5,82465	5,80456	0,440862	1,26296	0,73399	1,861029	0,48940645	0,0327109	0,316074737	0,00743015	ADP ribosylation factor like GTPase 14 effector protein like
ARNTL2	9,43933	6,93523	0,350482	0,675	0,5011	0,571367	0,34625746	0,04024127	0,058196219	0,00671395	aryl hydrocarbon receptor nuclear translocator like 2
ATF3	8,59937	8,50103	0,493973	0,69405	0,78697	1,481817	0,19340061	0,02523982	0,109569889	0,00279908	activating transcription factor 3
ATP6V0A1	13,97	13,491	0,40627	0,64474	0,37062	0,51116	0,26096821	0,0327109	0,094941587	0,00628738	ATPase H+ transporting V0 subunit a1
B3GALT4	8,18504	7,92608	-0,70317	-1,1176	-0,3003	-0,69343	0,13271469	0,00616978	0,477495378	0,03083865	beta-1,3-galactosyltransferase 4
BAG3	10,9889	8,22647	0,589476	0,70954	1,14098	2,51143	0,17657679	0,03557994	0,270457439	0,00816532	BAG cochaperone 3
BMPR1B-DT	7,11984	6,60847	-0,37172	-0,7419	-0,4022	-0,68715	0,32056332	0,02829988	0,257357275	0,01639477	BMPR1B divergent transcript
BSPRY	6,54611	6,56362	0,363272	0,81137	0,83502	1,196363	0,40185315	0,03977864	0,039144302	0,00212547	B-box and SPRY domain containing
C1GALT1C1L	7,67085	8,80966	-0,74093	-0,7936	-0,3819	-0,79115	0,0994574	0,02511396	0,257357275	0,00888423	C1GALT1 specific chaperone 1 like
CCDC114	6,86796	6,28499	0,993336	0,98287	0,39254	0,564549	0,11948799	0,0389095	0,298729724	0,03733401	coiled-coil domain containing 114
CCDC172	10,1805	8,63832	1,171962	1,59039	1,67129	2,058665	0,0012553	8,14E-05	0,038765528	0,0036294	coiled-coil domain containing 172
CD300H	8,83723	7,0801	3,284636	3,44524	1,66751	1,817258	2,79E-06	1,77E-06	0,040073161	0,00546503	CD300H molecule (gene/pseudogene)
CD68	10,6601	7,58488	0,499695	0,81381	1,28649	1,903889	0,27902454	0,03500513	0,001055715	3,38E-05	CD68 molecule
CDK5RAP1	10,5122	9,13463	-0,24066	-0,5437	-0,4158	-0,62399	0,3407205	0,02199979	0,172248401	0,01186799	CDK5 regulatory subunit associated protein 1
CYP2F1	6,0331	5,84622	0,34569	0,87363	0,6561	0,840717	0,36636851	0,01890267	0,163404333	0,01733298	cytochrome P450 family 2 subfamily F member 1
DDIT3	9,19152	9,38797	1,332211	1,91367	1,21572	2,455282	0,07021702	0,00383437	0,162584888	0,00424609	DNA damage inducible transcript 3
DDX46	10,0085	8,59022	-0,41267	-0,6033	-0,5542	-0,69963	0,19340061	0,02217473	0,103197099	0,00894825	DEAD-box helicase 46
DEDD2	12,3234	10,7917	0,352989	0,80418	0,47048	1,296863	0,26910382	0,00778299	0,524182085	0,02560877	death effector domain containing 2

DNAJB1	12,5639	11,963	0,561486	0,99734	1,56312	3,155849	0,14206236	0,003901	0,167276212	0,00439305	DnaJ heat shock protein family (Hsp40) member B1
DNAJB5	8,23636	10,008	0,352096	0,66171	0,25406	0,575358	0,3111757	0,03259979	0,39593224	0,01823015	DnaJ heat shock protein family (Hsp40) member B5
EDN2	11,1651	6,51213	0,674823	0,91235	1,39508	1,938042	0,10425032	0,01032244	0,000904855	3,84E-05	endothelin 2
FABP6	7,24995	7,03053	0,394372	0,56824	0,84057	1,067104	0,25506284	0,04290565	0,090123014	0,00743705	fatty acid binding protein 6
FAM189A1	7,09784	6,48904	0,514335	0,90614	0,46079	0,724709	0,27305481	0,02509487	0,348809723	0,03996744	family with sequence similarity 189 member A1
FAM189A2	6,22495	5,59012	0,918262	0,90054	0,56602	0,775899	0,03140825	0,01262777	0,13764021	0,01068263	family with sequence similarity 189 member A2
FAM241B	12,1121	11,7745	0,394992	0,65327	0,4647	1,083187	0,27927499	0,03370483	0,49658794	0,03516864	family with sequence similarity 241 member B
FICD	9,43182	8,73941	0,359093	0,76054	0,76181	2,041396	0,19798177	0,00394383	0,444240071	0,01567391	FIC domain protein adenylyltransferase
FKBP4	11,8773	10,8975	0,515058	0,61783	0,51915	0,792933	0,16911639	0,03238816	0,095562778	0,00479245	FKBP prolyl isomerase 4
FOSB	7,77225	7,3099	1,662022	2,66553	1,72529	3,417194	0,00239558	4,57E-05	0,137938689	0,0035287	FosB proto-oncogene, AP-1 transcription factor subunit
GADD45A	11,4389	10,8451	0,629285	0,82954	1,09979	1,772706	0,07354783	0,00662417	0,011637009	0,00036423	growth arrest and DNA damage inducible alpha
GBAP1	10,0248	10,156	0,532472	0,90422	0,58083	1,087284	0,12146093	0,003423	0,294655979	0,01646497	glucosylceramidase beta pseudogene 1
GBP5	6,03215	6,12497	0,568276	1,0082	0,40709	0,646604	0,25729389	0,02190384	0,3556904	0,04024728	guanylate binding protein 5
GCAT	8,15205	9,98519	0,692826	0,76481	0,44343	0,557113	0,05535032	0,01218963	0,153987455	0,01684129	glycine C-acetyltransferase
GCNA	7,55521	8,15061	0,871353	1,01006	1,81041	3,64563	0,14198852	0,02888374	0,205626421	0,00623591	germ cell nuclear acidic peptidase
GEM	8,76746	9,37942	0,417902	1,10301	0,40662	1,229088	0,35886734	0,01424272	0,549638774	0,02263003	GTP binding protein overexpressed in skeletal muscle
GOT1	12,88	11,0309	0,456389	0,66533	0,2196	0,614082	0,17182001	0,01648827	0,401219716	0,00989026	glutamic-oxaloacetic transaminase 1
GPR160	8,66769	6,84358	0,803239	1,03627	0,51389	0,517258	0,20073095	0,03876071	0,114006038	0,02079916	G protein-coupled receptor 160
H19	8,52441	9,09612	-1,10998	-1,3837	0,77467	0,802156	0,08158214	0,01142859	0,169465261	0,03447285	H19 imprinted maternally expressed transcript
HMOX1	16,936	14,0676	1,395134	1,56228	5,27027	6,810351	0,00369027	0,00086699	0,012136562	0,00096511	heme oxygenase 1
HRG	6,40505	5,92607	0,373837	0,91355	0,45738	1,196013	0,34625746	0,01728295	0,436773546	0,01572915	histidine rich glycoprotein
HSD17B6	6,91319	7,96387	0,804972	1,09873	0,64459	0,912761	0,19040613	0,02659639	0,052427798	0,00314237	hydroxysteroid 17-beta dehydrogenase 6
HSPA1B	14,2824	13,9286	1,432675	2,04958	2,14069	3,728357	0,0072321	0,0003531	0,115337447	0,00395575	heat shock protein family A (Hsp70) member 1B

HSPA1L	7,11168	7,51968	0,616348	1,04176	0,63246	1,470484	0,10425032	0,00288024	0,452880231	0,02533906	heat shock protein family A (Hsp70) member 1 like
HSPH1	13,8537	13,0183	0,515464	0,6959	0,35189	1,177627	0,20394935	0,03493424	0,652015284	0,03489515	heat shock protein family H (Hsp110) member 1
JMJD1C-AS1	8,04513	8,93591	-0,31145	-0,6677	-0,3204	-0,58366	0,26683194	0,01054308	0,362900956	0,02824613	JMJD1C antisense RNA 1
KIF25-AS1	6,95035	6,53903	-0,22764	-0,5266	-0,2194	-0,54643	0,42212218	0,04085257	0,508864894	0,03130969	KIF25 antisense RNA 1
KLHDC8A	6,94421	6,33374	0,496314	0,78406	0,73272	1,396348	0,26683194	0,03500513	0,02722859	0,00041057	kelch domain containing 8A
KLHL18	10,0934	10,0279	0,499803	0,75468	0,26659	0,780351	0,21847955	0,02858277	0,54853806	0,02465242	kelch like family member 18
LAMA5-AS1	10,7471	9,96561	0,378001	0,88864	0,27724	0,526126	0,42212218	0,0389095	0,447759564	0,04431753	LAMA5 antisense RNA 1
LINC00624	5,94153	6,62562	0,74056	1,21049	0,94273	2,284893	0,01583387	0,00033115	0,283839552	0,00682952	long intergenic non-protein coding RNA 624
LINC01201	8,42193	6,58252	-0,86775	-1,0368	-0,2454	-0,6043	0,00855707	0,00130249	0,42373956	0,01734876	long intergenic non-protein coding RNA 1201
LINC01511	7,42213	6,16816	1,283418	2,01637	0,64009	1,382401	0,00239558	5,36E-05	0,386907788	0,01979143	long intergenic non-protein coding RNA 1511
LINC01963	9,50639	9,58803	-0,73446	-0,9535	-0,4929	-0,63174	0,2184559	0,0451969	0,114279552	0,00989026	long intergenic non-protein coding RNA 1963
LINC02543	7,97671	5,87213	0,682324	0,83235	0,48725	0,598588	0,08158214	0,01218963	0,157244939	0,01849187	long intergenic non-protein coding RNA 2543
LOC10050669	8,40627	7,42059	0,340723	0,58194	0,55506	0,863136	0,27784371	0,02888374	0,037977038	0,00125484	uncharacterized LOC100506691
LOC10537036	6,48639	5,51211	0,532906	1,28997	0,51807	0,980534	0,31451992	0,01249787	0,344499038	0,0225721	uncharacterized LOC105370364
LOC10537210	10,8825	9,16343	3,293948	3,727	2,15334	2,442802	1,42E-05	3,91E-06	0,004171688	0,00055863	uncharacterized LOC105372100
LOC10798689	7,1127	7,52059	0,626547	0,6199	0,63965	0,651574	0,14206236	0,04770881	0,03789791	0,00601793	uncharacterized LOC107986898
LY96	6,40417	5,89721	0,876211	1,40444	0,73746	1,870592	0,22573133	0,02478063	0,338484603	0,00857921	lymphocyte antigen 96
MAP1LC3B	10,8124	10,5403	0,513068	1,07653	0,3646	0,857735	0,28916071	0,01693596	0,396245276	0,01639477	microtubule associated protein 1 light chain 3 beta
MBOAT4	5,74957	5,78	0,313649	0,54349	0,67928	0,834567	0,28003399	0,03080527	0,118866728	0,01234331	membrane bound O-acyltransferase domain containing 4
MEIG1	7,40105	7,24896	-0,20018	-0,5606	-0,4918	-0,62612	0,45272752	0,02788365	0,098663737	0,00838222	meiosis/spermiogenesis associated 1
MRM1	10,3734	8,23593	-0,55189	-0,7578	-0,3425	-0,734	0,19151099	0,02659639	0,42747929	0,02698231	mitochondrial rRNA methyltransferase 1
MSRA	11,0421	9,88812	-0,36119	-0,5733	-0,4368	-0,56627	0,28916071	0,04349884	0,150408479	0,01459939	methionine sulfoxide reductase A
MSX1	8,55649	5,91761	0,55747	0,79818	0,68624	1,49281	0,17396204	0,01773096	0,285003273	0,00950812	msh homeobox 1
MT1HL1	16,8526	15,7539	3,64044	3,95617	6,42206	6,597955	8,46E-07	5,08E-07	1,33E-08	5,29E-09	metallothionein 1H like 1
MYOM2	8,54803	7,21537	0,347461	0,71716	0,33624	0,903776	0,37816308	0,04290565	0,545987254	0,03142621	myomesin 2

NEK3	7,93439	8,10209	-0,8621	-1,327	-0,5926	-1,14065	0,04244146	0,00130249	0,352367679	0,0225721	NIMA related kinase 3
NEURL3	8,29136	5,6234	0,9179	1,16732	0,53927	1,246865	0,19493267	0,03730155	0,295171066	0,00842251	neuralized E3 ubiquitin protein ligase 3
OR8B8	9,38764	8,36667	0,343522	0,65173	0,67576	1,082922	0,34916665	0,04323215	0,214181857	0,01359952	olfactory receptor family 8 subfamily B member 8
PDE7B	7,88554	8,00562	-0,36217	-0,6762	-0,5764	-0,83878	0,32994278	0,03876071	0,235794674	0,02205452	phosphodiesterase 7B
PEG13	6,38706	7,17891	0,542358	0,71721	-0,2843	-0,63572	0,19327779	0,03086969	0,507081398	0,04203412	paternally expressed 13
PGBD2	8,82297	8,75898	0,48733	0,51302	0,34944	0,563817	0,12146093	0,03270792	0,163404333	0,00840142	piggyBac transposable element derived 2
PLA2G4C	8,89293	7,14849	1,723427	2,11261	1,02122	1,628894	0,0011846	0,00015752	0,091646423	0,00385192	phospholipase A2 group IVC
PLAC1	7,32106	5,85324	0,79429	1,19364	0,43065	0,814948	0,023821	0,00082583	0,290697089	0,01567391	placenta enriched 1
PLEK2	10,283	6,23331	0,341002	0,65296	0,85349	1,387608	0,30448382	0,02888374	0,037977038	0,00102735	pleckstrin 2
PLTP	7,77242	11,2778	0,567165	1,27758	0,5899	0,782133	0,3117212	0,01700195	0,056358013	0,00413165	phospholipid transfer protein
PRRG2	6,92276	6,63223	0,282735	0,63819	0,70695	1,4769	0,41909423	0,04290565	0,391518413	0,02303087	proline rich and Gla domain 2
RASIP1	8,31169	8,73666	0,388496	0,72349	0,40087	0,810866	0,35862389	0,04848818	0,166758566	0,00434937	Ras interacting protein 1
RCOR2	9,03866	12,4427	-0,55156	-0,7664	-0,2955	-0,7308	0,24471985	0,04373342	0,233075826	0,0041987	REST corepressor 2
RFPL2	6,46782	5,83778	0,750557	1,5002	0,3902	1,107013	0,17396204	0,00312557	0,586731038	0,03552943	ret finger protein like 2
RIPK4	8,68328	5,99103	0,394892	0,52256	0,34721	0,593555	0,19493267	0,0327109	0,229412531	0,0127569	receptor interacting serine/threonine kinase 4
RTL5	9,4717	12,0735	-0,40916	-0,7783	-0,3967	-0,82435	0,18627714	0,00538389	0,313275469	0,01359952	retrotransposon Gag like 5
RTL8B	8,1813	9,73043	-0,26654	-0,5388	-0,2426	-0,54044	0,37571437	0,04368007	0,274637232	0,00816532	retrotransposon Gag like 8B
SESN2	8,12369	7,77349	0,372351	0,62817	0,67137	1,484973	0,26085751	0,02696998	0,312934847	0,01127408	sestrin 2
SLC30A1	11,8598	10,121	1,57019	1,83258	2,12894	2,263922	7,08E-05	1,66E-05	3,90E-05	1,20E-05	solute carrier family 30 member 1
SLC30A2	8,28121	8,12345	2,832107	3,0745	3,1799	3,677543	3,21E-05	1,48E-05	0,009270457	0,00101022	solute carrier family 30 member 2
SMAD7	9,97619	9,76213	0,439066	0,72723	0,21913	0,603817	0,2339901	0,02326297	0,332408142	0,00651257	SMAD family member 7
SOX8	8,07797	6,54665	0,399659	1,01575	0,57146	1,545417	0,35586249	0,01662479	0,388378815	0,00989026	SRY-box transcription factor 8
SSUH2	7,74271	9,45476	0,552772	0,83817	0,90773	1,365486	0,27784371	0,04290565	0,014377461	0,00060344	ssu-2 homolog
SUSD5	6,43947	5,98393	1,075821	1,25602	0,45629	0,687439	0,17396204	0,03741622	0,263821374	0,02533906	sushi domain containing 5
TEX19	8,41193	9,90876	1,356742	2,03956	2,45944	3,700725	0,00847506	0,00031577	0,112730967	0,00601793	testis expressed 19
TMEM41B	11,0372	9,79041	0,466982	0,71195	0,32287	0,868477	0,21136171	0,02507731	0,458642184	0,01673291	transmembrane protein 41B
TMEM79	10,0488	9,51774	0,566431	0,898	0,27862	0,884074	0,0793015	0,00280312	0,640696317	0,03681392	transmembrane protein 79
TNFRSF8	7,17036	6,74922	0,680781	1,05958	0,71374	1,421483	0,17396204	0,01262777	0,355933631	0,02061803	TNF receptor superfamily member 8
TTC9B	7,10987	11,9906	0,954766	1,6986	1,35645	2,106556	0,04892893	0,00059438	0,101553983	0,00479771	tetratricopeptide repeat domain 9B

UBXN8	10,2325	9,61775	0,549141	0,79187	0,2655	0,603441	0,12146093	0,0086919	0,477637532	0,0322335	UBX domain protein 8
UQCC3	14,924	12,7659	-0,28208	-0,6259	-0,5515	-0,93386	0,36717409	0,03128412	0,153754174	0,00667727	ubiquinol-cytochrome c reductase complex assembly factor 3
ZFAND2A	11,6637	11,2703	0,694674	1,20636	2,16884	4,06832	0,1037185	0,00233856	0,132213575	0,00383099	zinc finger AN1-type containing 2A
ZNF143	9,76289	8,70486	0,440073	0,61171	0,29567	0,689074	0,25161944	0,04590229	0,365175532	0,0136595	zinc finger protein 143
ZNF165	9,82852	8,88812	1,669699	2,62201	1,12778	2,088581	0,00012578	3,15E-06	0,281215563	0,01571098	zinc finger protein 165
ZNF350	9,00342	8,16847	-0,23672	-0,6127	-0,5139	-0,71859	0,37321231	0,01873425	0,082104549	0,0052742	zinc finger protein 350
