# Supplementary material for: Is Cadmium Toxicity Tissue-Specific? Toxicogenomics Studies Reveal Common and Specific Pathways in Pulmonary, Hepatic, and Neuronal Cell Models
Source: Int J Mol Sci. 2022 Feb 4;23(3):1768. doi: 10.3390/ijms23031768 (PMC8836438; doi:10.3390/ijms23031768)
Supplement: Supplementary file 1 [file ijms-23-01768-s001.zip › Table S3.pdf]

Table S3. Comparison of regulated genes (up and down) in common in pulmonary (A549), and hepatic (HepG2) cell models exposed to Cd for 24 hours.

Gene	AveExpr. HEPG2	AveExpr. A549	logFC.2. HEPG2	logFC.10. HEPG2	logFC.10. A549	logFC.20. A549	adj.P.Val.2. HEPG2	adj.P.Val.10 .HEPG2	adj.P.Val.10 .A549	adj.P.Val.20. A549	Description
SERPINE1	9,06	9,02	0,61	2,77	0,89	1,26	0,9884185	0,00212986	0,17001949	0,01773096	serpin family E member 1
METRNL	10,95	8,45	0,74	2,46	0,66	0,73	0,6308422	0,00046514	0,03312847	0,00721744	meteorin like, glial cell differentiation regulator
RND3	7,44	11,08	0,22	2,32	0,40	0,68	0,9997134	0,01326343	0,27927499	0,03247194	Rho family GTPase 3
TGM2	10,52	10,80	0,73	2,19	0,71	1,00	0,7483777	0,00133462	0,1350925	0,01218963	transglutaminase 2
PSG6	6,12	5,91	0,36	2,00	0,46	0,79	0,9997134	0,00288259	0,3111757	0,04324383	pregnancy specific beta-1-glycoprotein 6
KRT15	9,91	11,26	0,39	1,82	0,53	0,91	0,7476439	0,00027361	0,21388124	0,01709219	keratin 15
MPZL3	11,40	6,47	0,45	1,75	0,32	0,56	0,9997134	0,02058221	0,27927499	0,02767496	myelin protein zero like 3
IL23A	8,10	7,77	0,27	1,69	0,70	1,06	0,9997134	0,00167105	0,07657072	0,00313627	interleukin 23 subunit alpha
MCL1	11,04	10,41	0,49	1,36	0,55	0,89	0,6623391	0,00113425	0,21683076	0,0205932	MCL1 apoptosis regulator, BCL2 family member
VGF	7,14	11,22	0,22	1,30	0,36	0,86	0,9997134	0,00411487	0,29872767	0,01086282	VGF nerve growth factor inducible
ZNF556	9,94	5,79	0,38	1,19	0,51	0,95	0,9303207	0,0047146	0,28478006	0,02511396	zinc finger protein 556
TM4SF19	5,65	8,73	0,48	1,11	0,73	1,16	0,7862474	0,00501327	0,08448537	0,00304855	transmembrane 4 L six family member 19
FAM135A	8,00	7,51	0,40	0,98	0,37	0,54	0,7898033	0,00403897	0,26141194	0,04331206	family with sequence similarity 135 member A
ITPRIPL2	10,58	11,51	0,27	0,94	-0,42	-0,53	0,9296766	0,00288259	0,14256381	0,02217473	ITPRIP like 2
PSPN	7,67	9,23	0,43	0,94	0,56	0,59	0,7431268	0,00460293	0,14256381	0,03971511	persephin
IL32	12,37	7,71	0,30	0,92	0,25	0,76	0,9997134	0,01881695	0,5579669	0,04398593	interleukin 32
VNN1	6,01	5,78	0,23	0,90	0,45	0,70	0,9869505	0,0037962	0,28916071	0,04644295	vanin 1
SCGB3A2	9,42	9,08	0,24	0,86	-0,38	-0,57	0,888547	0,00185752	0,19798177	0,02312812	secretoglobin family 3A member 2
STX3	9,65	8,15	0,24	0,83	0,55	0,76	0,9340848	0,00308607	0,12547651	0,01218963	syntaxin 3
MLF1	6,23	9,06	0,39	0,76	0,42	0,64	0,7483777	0,00880052	0,28150507	0,04803652	myeloid leukemia factor 1
LCP1	8,27	5,98	0,24	0,70	0,66	1,00	0,9846753	0,01401819	0,19327779	0,01892154	lymphocyte cytosolic protein 1
TNFRSF10D	6,87	11,71	0,35	0,60	0,59	0,85	0,7232364	0,01152233	0,15080498	0,01337906	TNF receptor superfamily member 10d
ZNF219	14,00	11,39	-0,26	-0,60	-0,50	-0,73	0,8743152	0,01164713	0,13180294	0,00950642	zinc finger protein 219
ZNF57	9,72	8,09	-0,29	-0,61	0,44	0,68	0,8703199	0,01474104	0,22056397	0,02715204	zinc finger protein 57
SEPHS2	13,35	12,60	-0,23	-0,69	0,64	0,60	0,9997134	0,03834416	0,03509569	0,01728295	selenophosphate synthetase 2
CD14	7,20	10,46	-0,33	-0,72	-0,66	-0,66	0,7431268	0,00470367	0,1401483	0,04368007	CD14 molecule
HSD17B7	9,90	7,42	-0,40	-1,06	0,45	0,54	0,875209	0,00624361	0,19798177	0,04375132	hydroxysteroid 17-beta dehydrogenase 7
AFMID	10,36	8,02	-0,30	-1,14	0,31	0,61	0,9303207	0,00194641	0,32597486	0,02985156	arylformamidase

PAQR8	9,45	6,94	-0,29	-1,15	-0,45	-0,58	0,9997134	0,01394168	0,20145951	0,03977864	progestin and adipoQ receptor family member 8
A1BG	8,24	8,45	-0,34	-1,20	0,56	0,72	0,9609636	0,00421207	0,08158214	0,00878566	alpha-1-B glycoprotein
CCDC142	9,54	9,41	-0,50	-1,24	0,34	0,53	0,9120202	0,0110313	0,22542515	0,02843037	coiled-coil domain containing 142
C4BPB	13,44	6,95	-0,44	-1,39	0,77	0,96	0,9028059	0,00345601	0,19327779	0,03783895	complement component 4 binding protein beta
PCSK9	12,57	11,00	-0,62	-2,39	-0,46	-0,78	0,9854096	0,00403897	0,2184559	0,01867886	proprotein convertase subtilisin/kexin type 9
