# Supplementary material for: Is Cadmium Toxicity Tissue-Specific? Toxicogenomics Studies Reveal Common and Specific Pathways in Pulmonary, Hepatic, and Neuronal Cell Models
Source: Int J Mol Sci. 2022 Feb 4;23(3):1768. doi: 10.3390/ijms23031768 (PMC8836438; doi:10.3390/ijms23031768)
Supplement: Supplementary file 1 [file ijms-23-01768-s001.zip › Table S4.pdf]

Table S4. Comparison of regulated genes (up and down) in common in hepatic (HepG2) and neuronal (SH-SY5Y) cell models exposed to Cd for 24 hours.

Gene	AveExpr. HEPG2	AveExpr. SHSY5Y	logFC.2. HEPG2	logFC.10. HEPG2	logFC.10. SHSY5Y	logFC.20 .SHSY5Y	adj.P.Val.2 .HEPG2	adj.P.Val.10 .HEPG2	adj.P.Val.10. SHSY5Y	adj.P.Val.20. SHSY5Y	Description
RRAD	9,78	8,67	1,38	4,77	3,27	5,42	0,2586251	9,59E-05	0,01895891	0,00054007	RRAD, Ras related glycolysis inhibitor and calcium channel regulator
CRYAB	9,93	8,85	2,34	3,75	3,19	4,90	0,5822455	0,00651583	0,02969749	0,00102735	crystallin alpha B
GDF15	16,33	13,24	0,68	1,64	3,88	4,39	0,3332181	0,00035957	1,28E-05	2,70E-06	growth differentiation factor 15
PHLDA2	11,92	8,99	0,85	2,27	2,76	3,63	0,624695	0,00085255	0,00205452	0,00014817	pleckstrin homology like domain family A member 2
EGR1	10,83	10,89	1,71	3,03	2,22	3,43	0,4170229	0,00165712	0,00495221	0,00014299	early growth response 1
EGR4	7,71	7,06	1,16	3,17	1,56	3,08	0,4122072	0,00029952	0,1031971	0,00216855	early growth response 4
S100A2	10,74	8,82	0,63	2,49	1,43	2,55	0,9997134	0,01418106	0,17283823	0,00677338	S100 calcium binding protein A2
TNFRSF12A	13,61	11,60	0,55	1,84	1,67	2,45	0,7431268	0,00080162	0,00114454	3,81E-05	TNF receptor superfamily member 12A
VCX2	6,68	7,47	0,61	1,46	1,12	2,44	0,4122072	0,00049847	0,27033364	0,00836725	variable charge X-linked 2
S100A16	12,84	9,51	0,38	1,45	1,93	2,32	0,9781613	0,00376695	0,00021203	2,58E-05	S100 calcium binding protein A16
CCR7	6,81	6,57	0,36	2,50	1,09	2,18	0,9997134	0,00215558	0,31306989	0,01567391	C-C motif chemokine receptor 7
CTAG1A	6,46	6,65	0,35	1,22	1,28	2,18	0,7431268	0,00067429	0,15227671	0,0064615	cancer/testis antigen 1A
CDKN1A	10,94	8,24	0,41	1,25	1,74	2,13	0,6308422	0,00056573	0,00015113	1,59E-05	cyclin dependent kinase inhibitor 1A
DUSP13	11,33	6,38	0,50	1,30	1,42	2,13	0,5163662	0,00059822	0,07104082	0,0036338	dual specificity phosphatase 13
FOS	7,02	7,43	1,03	3,36	1,08	2,12	0,7431268	0,00077032	0,1380698	0,0036294	Fos proto-oncogene, AP-1 transcription factor subunit
SERTAD1	12,48	11,48	0,27	1,35	1,06	1,93	0,9457244	0,00077213	0,09232403	0,00251419	SERTA domain containing 1
FTCD	12,07	6,81	-0,33	-0,92	0,97	1,86	0,9830101	0,01586045	0,17472191	0,00550642	formimidoyltransferase cyclodeaminase
ASB2	10,62	6,30	1,09	3,22	0,73	1,76	0,8066405	0,00190336	0,39911533	0,01532287	ankyrin repeat and SOCS box containing 2
HSPB2	5,95	6,00	0,40	0,97	0,87	1,71	0,8281899	0,00585861	0,28121556	0,01289476	heat shock protein family B (small) member 2
CNN1	9,50	7,71	0,64	1,70	0,97	1,62	0,6308422	0,00103167	0,01802211	0,00044049	calponin 1
C1S	10,54	5,94	-0,52	-1,16	0,77	1,56	0,8930622	0,01559353	0,15375417	0,00385192	complement C1s
SQSTM1	9,40	11,77	0,22	0,59	0,81	1,54	0,9120202	0,00797432	0,13990746	0,00395575	sequestosome 1
SNAI1	8,85	11,39	0,65	3,37	0,82	1,52	0,6364837	9,60E-05	0,23858724	0,0109698	snail family transcriptional repressor 1
NUPR1	13,83	6,81	-0,68	-1,08	0,61	1,52	0,8066405	0,02966169	0,48680701	0,02743746	nuclear protein 1, transcriptional regulator
CLDN4	9,33	7,47	0,26	1,07	0,97	1,44	0,9869505	0,00295104	0,03884881	0,00174909	claudin 4
S100A10	13,85	10,85	0,24	0,91	1,21	1,42	0,9730186	0,0034463	0,09556278	0,010811	S100 calcium binding protein A10
ARC	6,66	8,98	0,95	3,88	0,57	1,40	0,4610304	9,60E-05	0,15233523	0,00179175	activity regulated cytoskeleton associated protein

SPINT2	8,13	6,79	0,37	1,83	0,77	1,39	0,9997134	0,00893247	0,02272901	0,00041057	serine peptidase inhibitor, Kunitz type 2
RASD1	10,58	7,79	0,80	2,26	0,80	1,38	0,2204806	0,00014455	0,1380698	0,0052742	ras related dexamethasone induced 1
IER3	12,96	6,60	1,01	1,67	0,96	1,35	0,5851437	0,00617095	0,09611115	0,00617159	immediate early response 3
AGT	15,74	6,91	-0,40	-1,25	1,20	1,34	0,643273	0,00065593	0,00121972	0,00021382	angiotensinogen
FHL2	13,98	8,49	0,56	2,37	0,58	1,33	0,901611	0,00099146	0,28112183	0,00763871	four and a half LIM domains 2
ICAM2	11,59	10,33	0,40	1,42	1,16	1,31	0,9997134	0,02118213	0,00205452	0,00036423	intercellular adhesion molecule 2
HBEGF	7,07	7,43	0,26	1,35	0,87	1,29	0,8639199	0,00033956	0,07843133	0,00410067	heparin binding EGF like growth factor
SDCBP2	7,05	6,83	0,37	1,21	0,66	1,28	0,9869505	0,0081627	0,23956246	0,00928233	syndecan binding protein 2
BIK	8,35	6,24	0,22	1,60	0,71	1,27	0,9997134	0,01613489	0,04935866	0,00105305	BCL2 interacting killer
FLNC	5,51	8,34	0,22	1,14	0,70	1,26	0,9997134	0,00199528	0,05668297	0,00123707	filamin C
KRT19	17,35	8,66	0,33	1,31	0,78	1,26	0,9997134	0,01049652	0,2770227	0,02329127	keratin 19
TXNRD1	10,78	8,73	0,21	0,83	0,82	1,24	0,9997134	0,0359167	0,17080065	0,01126703	thioredoxin reductase 1
PROCR	10,79	7,38	0,34	1,93	0,95	1,23	0,9997134	0,00300426	0,01492759	0,0011348	protein C receptor
ZFP36	9,11	9,27	0,71	1,90	0,66	1,21	0,8066405	0,00301287	0,08882025	0,00220445	ZFP36 ring finger protein
SAT1	14,89	7,38	0,71	1,45	0,65	1,21	0,9120202	0,02443531	0,06310594	0,00123707	spermidine/spermine N1-acetyltransferase 1
IL15	6,57	8,06	0,25	0,57	1,01	1,21	0,9591153	0,02895043	0,00980225	0,00096511	interleukin 15
TAGLN	12,61	7,02	0,45	1,37	0,60	1,18	0,6933351	0,00082549	0,03119668	0,00041057	transgelin
GABARAPL1	8,80	8,42	0,24	1,34	0,71	1,16	0,94953	0,00049847	0,13928209	0,00628738	GABA type A receptor associated protein like 1
ACTA1	13,48	6,30	1,15	2,14	0,40	1,14	0,875209	0,02758283	0,34595156	0,00652876	actin alpha 1, skeletal muscle
MVP	13,93	10,77	0,33	1,14	0,96	1,13	0,8639199	0,00146913	0,00705371	0,00076972	major vault protein
RELB	12,22	10,01	0,42	1,62	0,91	1,12	0,7431268	0,00048298	0,03876553	0,0036338	RELB proto-oncogene, NF-kB subunit
FOSL1	7,79	6,48	0,55	1,24	0,32	1,11	0,8257088	0,00770497	0,69698571	0,04482117	FOS like 1, AP-1 transcription factor subunit
S100A11	10,30	11,18	0,45	1,68	0,72	1,11	0,9997134	0,02805579	0,06333648	0,00291644	S100 calcium binding protein A11
PLK3	12,10	10,21	0,42	1,31	0,79	1,10	0,9591153	0,00758166	0,0135679	0,00082276	polo like kinase 3
SPOCD1	6,76	5,81	0,41	1,79	0,82	1,10	0,6976716	0,00023525	0,00678713	0,00041057	SPOC domain containing 1
TIMP1	10,02	10,31	0,60	1,53	0,90	1,08	0,6189363	0,00099251	0,0135679	0,00141522	TIMP metallopeptidase inhibitor 1
MAFF	8,41	7,27	0,58	1,96	0,43	1,03	0,7483777	0,00081789	0,19969548	0,00353386	MAF bZIP transcription factor F
UPP1	8,26	9,17	0,49	0,99	0,68	1,02	0,7862474	0,00981541	0,04935866	0,00216855	uridine phosphorylase 1
ACTG2	7,91	6,63	0,26	1,34	0,61	1,01	0,9997134	0,02065803	0,23967716	0,01538857	actin gamma 2, smooth muscle
TUBA4A	12,09	8,43	0,22	1,16	0,53	1,01	0,9997134	0,019689	0,34030334	0,02201381	tubulin alpha 4a
SRXN1	13,25	11,74	0,35	0,85	0,49	0,99	0,9248016	0,01278852	0,32115961	0,01571098	sulfiredoxin 1

SERPINE2	11,22	6,52	0,84	2,37	0,32	0,98	0,9591153	0,01100556	0,40177006	0,00767612	serpin family E member 2
NPPB	9,36	6,01	0,99	3,13	0,50	0,98	0,8066405	0,00141847	0,19349796	0,00601793	natriuretic peptide B
CILP	5,62	6,54	0,26	1,57	0,74	0,98	0,9997134	0,00212986	0,02427443	0,00165812	cartilage intermediate layer protein
IL6R	8,86	5,96	0,22	0,98	0,42	0,96	0,9997134	0,00417584	0,20716531	0,00410067	interleukin 6 receptor
MAFK	8,02	8,51	0,23	0,70	0,56	0,94	0,9464353	0,00674754	0,09177614	0,00324922	MAF bZIP transcription factor K
HTRA3	6,61	5,84	0,60	2,44	0,46	0,92	0,5822445	0,00014455	0,40177006	0,02817497	HtrA serine peptidase 3
CCK	6,31	5,70	0,48	2,10	0,47	0,92	0,9333199	0,00106016	0,18485161	0,00573815	cholecystokinin
OSBPL10	9,36	6,86	0,21	1,06	0,69	0,90	0,9997134	0,00463047	0,11321801	0,00894825	oxysterol binding protein like 10
APOH	16,66	5,58	-0,23	-1,28	0,65	0,90	0,9997134	0,00305542	0,08200806	0,0052742	apolipoprotein H
MICALL2	10,11	10,67	0,33	0,85	0,59	0,89	0,8639199	0,00599455	0,0905736	0,00444026	MICAL like 2
S100A3	9,06	5,88	0,30	2,13	0,67	0,89	0,9997134	0,02430607	0,04686709	0,00342679	S100 calcium binding protein A3
EPHA2	10,14	7,14	0,73	1,80	0,48	0,89	0,6507079	0,00172011	0,21726903	0,00867218	EPH receptor A2
RAB31	7,95	10,68	0,32	1,31	0,70	0,88	0,9444595	0,00150786	0,08402899	0,00713809	RAB31, member RAS oncogene family
EGR2	5,91	6,00	0,36	1,22	0,45	0,87	0,9740075	0,00576384	0,17125605	0,0052742	early growth response 2
MGLL	6,77	8,60	0,30	1,48	0,31	0,87	0,9997134	0,00490887	0,40933999	0,010811	monoglyceride lipase
TRPV2	12,61	7,21	0,30	1,64	0,63	0,86	0,9997134	0,00143437	0,03876553	0,00251419	transient receptor potential cation channel subfamily V member 2
CNN2	11,67	8,80	0,33	0,56	0,55	0,84	0,8814889	0,04057702	0,10340148	0,0050463	calponin 2
ABCB6	12,94	12,48	-0,21	-0,93	0,70	0,84	0,9997134	0,01836176	0,00980225	0,00096511	ATP binding cassette subfamily B member 6 (Langereis blood group)
GFPT2	6,91	7,68	0,44	2,36	0,41	0,84	0,9997134	0,00624361	0,1590817	0,00385192	glutamine-fructose-6-phosphate transaminase 2
CSRP1	8,98	7,60	0,24	0,67	0,65	0,83	0,9997134	0,01874646	0,01847893	0,00146799	cysteine and glycine rich protein 1
SCARA3	7,45	9,49	-0,33	-0,90	0,63	0,83	0,9997134	0,04295537	0,0135679	0,0009668	scavenger receptor class A member 3
SFN	10,46	6,87	0,40	0,76	0,33	0,81	0,906725	0,03225142	0,5533971	0,04359481	stratifin
FADS3	9,92	8,15	0,44	1,31	0,70	0,81	0,8066405	0,0018701	0,07818466	0,00846482	fatty acid desaturase 3
RAB7B	7,29	5,74	0,33	2,04	0,73	0,79	0,9235706	0,0002899	0,08477448	0,0121391	RAB7B, member RAS oncogene family
LHX6	7,46	6,79	0,36	1,61	0,53	0,78	0,8639199	0,00057385	0,076197	0,00416296	LIM homeobox 6
SH2B3	7,54	8,20	0,30	0,73	0,41	0,72	0,8181735	0,00489291	0,15040848	0,00546503	SH2B adaptor protein 3
ASS1	10,69	6,90	-0,47	-1,46	0,60	0,72	0,945262	0,00534665	0,06553296	0,00671395	argininosuccinate synthase 1
DUSP1	9,70	9,13	0,22	1,49	0,29	0,71	0,9997134	0,00167105	0,47776373	0,02701885	dual specificity phosphatase 1
NGFR	6,91	6,91	0,29	2,25	0,42	0,71	0,9997134	0,00933506	0,1312053	0,0052307	nerve growth factor receptor

TUBB6	14,98	14,07	0,38	1,02	0,72	0,71	0,9255985	0,00844006	0,0115899	0,00276249	tubulin beta 6 class V
NUAK2	9,28	6,65	0,26	1,08	0,30	0,70	0,9586893	0,00199528	0,2572015	0,00660702	NUAK family kinase 2
RAB3A	7,40	10,83	0,41	1,62	0,45	0,69	0,8232327	0,0006482	0,11957109	0,00652876	RAB3A, member RAS oncogene family
SLC6A12	6,91	6,98	-0,56	-1,89	0,40	0,68	0,7431268	0,00075298	0,1533403	0,00665803	solute carrier family 6 member 12
NPAS1	8,01	6,71	0,82	1,62	0,46	0,68	0,5732982	0,00207902	0,09029923	0,00485382	neuronal PAS domain protein 1
KRT80	7,28	7,56	0,28	1,59	0,27	0,68	0,9997134	0,00489826	0,23766575	0,00431466	keratin 80
LMTK2	6,54	8,98	0,29	0,70	0,34	0,68	0,9591153	0,02361637	0,2779374	0,0127569	lemur tyrosine kinase 2
TGFB1	9,31	7,84	0,48	0,88	0,58	0,67	0,6243907	0,00434725	0,0524278	0,00601793	transforming growth factor beta 1
PNPLA3	10,88	6,38	-0,64	-1,47	0,60	0,66	0,7431268	0,00403897	0,02474156	0,0036294	patatin like phospholipase domain containing 3
PREX1	6,69	9,11	0,47	1,49	0,55	0,64	0,7431268	0,00097174	0,04298745	0,00492635	phosphatidylinositol-3,4,5-trisphosphate dependent Rac exchange factor 1
MAP3K14	9,23	8,59	0,23	1,05	0,33	0,63	0,9997134	0,00682331	0,39803358	0,03030424	mitogen-activated protein kinase kinase kinase 14
PLXND1	9,75	9,01	0,38	1,38	0,56	0,61	0,9915628	0,00547814	0,04081613	0,00546391	plexin D1
ARRDC2	8,99	8,73	0,37	0,83	0,46	0,61	0,8097407	0,00690052	0,09185497	0,00671395	arrestin domain containing 2
KLF5	6,97	5,81	0,57	1,60	0,36	0,60	0,4610304	0,00033956	0,36597727	0,03674935	Kruppel like factor 5
PFKP	11,10	12,24	0,76	2,09	0,37	0,59	0,8106562	0,00295104	0,12552455	0,00580596	phosphofructokinase, platelet
SOCS1	7,73	6,45	-0,24	-0,63	0,29	0,59	0,9997134	0,04992086	0,39139346	0,02572144	suppressor of cytokine signaling 1
IL4R	14,44	5,79	0,39	1,27	0,40	0,59	0,9061084	0,00315166	0,19599764	0,01517863	interleukin 4 receptor
NTNG2	6,39	7,70	0,23	1,12	0,40	0,59	0,9997134	0,00213997	0,1359844	0,00816702	netrin G2
RHOB	9,52	9,26	0,38	0,81	0,32	0,57	0,7483777	0,00645887	0,20046848	0,00842251	ras homolog family member B
FBLIM1	9,39	8,37	0,45	1,72	0,23	0,56	0,9997134	0,00880052	0,27463723	0,00618685	filamin binding LIM protein 1
NR1D1	9,02	7,29	0,44	0,66	0,39	0,56	0,7431268	0,02527872	0,23744565	0,02305668	nuclear receptor subfamily 1 group D member 1
GAL3ST1	10,62	8,49	-0,27	-1,24	0,63	0,54	0,9997134	0,03607393	0,15040848	0,04763102	galactose-3-O-sulfotransferase 1
S100A6	14,05	12,90	0,30	1,85	0,30	0,54	0,9997134	0,02908325	0,18699216	0,00713597	S100 calcium binding protein A6
SEC14L1	9,49	10,50	0,25	0,94	0,23	0,54	0,9571375	0,00282804	0,46756516	0,02626713	SEC14 like lipid binding 1
ADORA2B	10,79	8,76	0,20	0,61	0,63	0,53	0,9997134	0,02908325	0,06304956	0,01817274	adenosine A2b receptor
CYP1A1	9,63	5,97	0,68	2,97	0,41	0,53	0,9997134	0,01113399	0,23967716	0,03168983	cytochrome P450 family 1 subfamily A member 1
C1orf115	10,39	6,46	-0,53	-1,22	0,55	0,53	0,6623391	0,00251659	0,05852524	0,01158566	chromosome 1 open reading frame 115
RGS2	8,18	12,03	0,59	2,67	0,40	0,52	0,7431268	0,00027927	0,12840094	0,01160481	regulator of G protein signaling 2
HSPB8	7,05	5,84	0,51	2,01	0,32	0,52	0,9457244	0,00201157	0,24105407	0,01646497	heat shock protein family B (small) member 8
TGFB1I1	7,56	11,98	0,52	1,94	0,41	0,52	0,9997134	0,00657	0,06751142	0,00601793	transforming growth factor beta 1 induced transcript 1

MLLT11	9,06	15,42	0,67	2,40	0,42	0,52	0,7550777	0,00069232	0,20058027	0,0264639	MLLT11 transcription factor 7 cofactor
PDZK1	10,92	8,46	-0,37	-0,68	0,24	0,52	0,928039	0,03978716	0,28121556	0,0093638	PDZ domain containing 1
GPR137B	6,83	8,37	0,35	1,29	0,22	0,51	0,9303207	0,00212986	0,34616837	0,01240204	G protein-coupled receptor 137B
LMNA	14,19	13,33	0,32	0,66	0,36	0,50	0,7483777	0,00690052	0,11533745	0,00726863	lamin A/C
CLCF1	7,68	5,99	0,45	1,58	0,33	0,50	0,7476439	0,00068055	0,2098929	0,01512167	cardiotrophin like cytokine factor 1
E2F8	9,22	7,53	-0,25	-1,70	-0,34	-0,52	0,9997134	0,00841078	0,3154619	0,0346684	E2F transcription factor 8
BIRC5	15,45	15,02	-0,26	-0,79	-0,24	-0,52	0,888547	0,0034463	0,2654518	0,00816702	baculoviral IAP repeat containing 5
CNNM3	10,76	9,27	-0,23	-0,65	-0,27	-0,52	0,9997134	0,044164	0,33391256	0,01955089	cyclin and CBS domain divalent metal cation transport mediator 3
DUSP6	11,19	8,68	0,88	1,91	-0,34	-0,53	0,4610304	0,00078647	0,2779374	0,02541825	dual specificity phosphatase 6
ACBD7	10,67	7,13	-0,42	-1,00	-0,29	-0,56	0,9351359	0,01558759	0,1766067	0,00597753	acyl-CoA binding domain containing 7
NUF2	11,85	10,73	-0,22	-1,15	-0,49	-0,57	0,9577383	0,00069232	0,0401191	0,00479245	NUF2 component of NDC80 kinetochore complex
MCCC1	12,81	10,32	-0,28	-0,72	-0,26	-0,58	0,8753346	0,00748987	0,34469874	0,01416034	methylcrotonoyl-CoA carboxylase 1
RCOR3	7,62	8,13	-0,20	-0,51	-0,34	-0,59	0,9997134	0,03781547	0,2770227	0,01877739	REST corepressor 3
FGD6	7,78	7,32	0,25	0,58	-0,43	-0,66	0,9457244	0,02259114	0,14940039	0,00799108	FYVE, RhoGEF and PH domain containing 6
METTL7A	9,41	12,45	-0,41	-2,15	-0,36	-0,66	0,6507079	0,00011212	0,18959492	0,00696542	methyltransferase like 7A
FBXO4	8,66	6,55	-0,39	-1,24	-0,31	-0,68	0,945262	0,00489873	0,29153408	0,00989026	F-box protein 4
VSNL1	11,64	7,34	-0,26	-0,94	-0,44	-0,70	0,9869505	0,00497206	0,31306989	0,03156015	visinin like 1
LOC644656	9,06	7,47	-0,32	-0,93	-0,54	-0,70	0,9997134	0,03092034	0,10920729	0,00866941	uncharacterized LOC644656
MFAP4	5,85	14,83	-0,28	-0,59	-0,36	-0,73	0,888547	0,01965087	0,13369348	0,00284995	microfibril associated protein 4
PRSS36	11,42	7,70	-0,52	-0,94	-0,70	-0,73	0,4091634	0,0014083	0,11191704	0,01817274	serine protease 36
CDCA3	11,15	10,48	-0,21	-1,18	-0,54	-0,74	0,9997134	0,00093548	0,1062807	0,00720604	cell division cycle associated 3
FOXN4	5,71	10,29	-0,32	-0,80	-0,48	-0,81	0,875209	0,00799835	0,25847407	0,01735726	forkhead box N4
CDKN2C	11,96	12,01	-0,57	-1,61	-0,53	-0,81	0,968072	0,01233867	0,05225786	0,00224508	cyclin dependent kinase inhibitor 2C
GLCCI1	9,64	10,56	0,59	2,16	-0,77	-0,86	0,9997134	0,02324292	0,01122615	0,00146799	glucocorticoid induced 1
PDGFRL	7,66	7,46	-0,31	-1,05	-0,38	-0,88	0,7862474	0,00104556	0,37905536	0,01572915	platelet derived growth factor receptor like
HLF	12,84	6,64	-0,23	-1,23	-0,55	-1,06	0,9997134	0,00415299	0,45915579	0,04526024	HLF transcription factor, PAR bZIP family member
