# Supplementary figures and images for: Is Cadmium Toxicity Tissue-Specific? Toxicogenomics Studies Reveal Common and Specific Pathways in Pulmonary, Hepatic, and Neuronal Cell Models
Source: Int J Mol Sci. 2022 Feb 4;23(3):1768. doi: 10.3390/ijms23031768 (PMC8836438; doi:10.3390/ijms23031768)

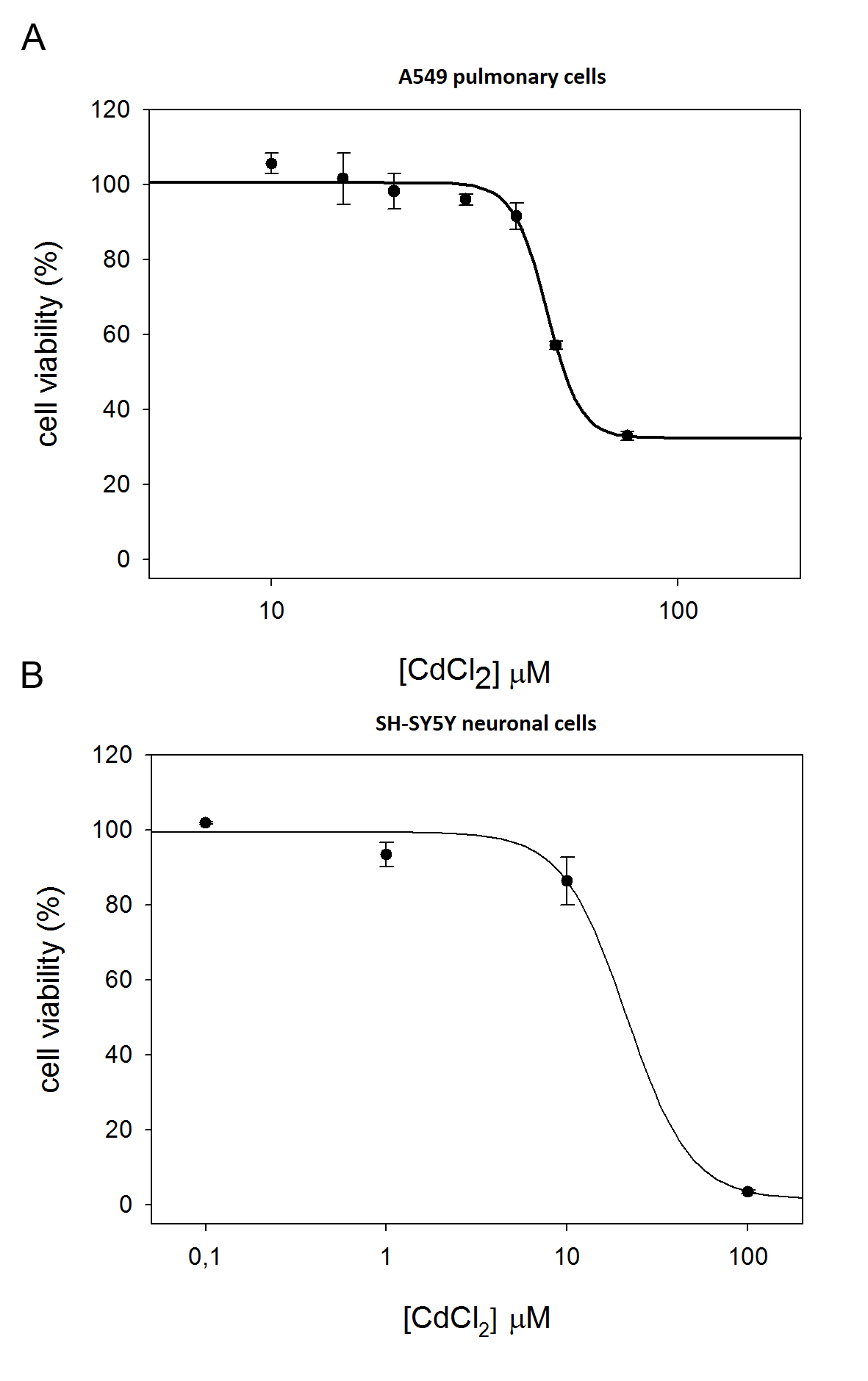

Supplement: Supplementary file 1 [file ijms-23-01768-s001.zip › Figure S1.tif]
